# mBrain: towards the continuous follow-up and headache classification of primary headache disorder patients

**DOI:** 10.1186/s12911-022-01813-w

**Published:** 2022-03-31

**Authors:** Mathias De Brouwer, Nicolas Vandenbussche, Bram Steenwinckel, Marija Stojchevska, Jonas Van Der Donckt, Vic Degraeve, Jasper Vaneessen, Filip De Turck, Bruno Volckaert, Paul Boon, Koen Paemeleire, Sofie Van Hoecke, Femke Ongenae

**Affiliations:** 1 IDLab, Ghent University – imec, 9052 Ghent, Belgium; 2 Department of Neurology, Ghent University Hospital, 9000 Ghent, Belgium; 3 4BRAIN, Institute for Neuroscience, Department of Head and Skin, Ghent University, 9000 Ghent, Belgium

**Keywords:** Headache classification, Continuous headache follow-up, Knowledge-based, Machine learning, Context-aware, Headache trigger detection, Semantics, Mobile application, Physiological wearable data, Primary headache disorder

## Abstract

**Background:**

The diagnosis of headache disorders relies on the correct classification of individual headache attacks. Currently, this is mainly done by clinicians in a clinical setting, which is dependent on subjective self-reported input from patients. Existing classification apps also rely on self-reported information and lack validation. Therefore, the exploratory mBrain study investigates moving to continuous, semi-autonomous and objective follow-up and classification based on both self-reported and objective physiological and contextual data.

**Methods:**

The data collection set-up of the observational, longitudinal mBrain study involved physiological data from the Empatica E4 wearable, data-driven machine learning (ML) algorithms detecting activity, stress and sleep events from the wearables’ data modalities, and a custom-made application to interact with these events and keep a diary of contextual and headache-specific data. A knowledge-based classification system for individual headache attacks was designed, focusing on migraine, cluster headache (CH) and tension-type headache (TTH) attacks, by using the classification criteria of ICHD-3. To show how headache and physiological data can be linked, a basic knowledge-based system for headache trigger detection is presented.

**Results:**

In two waves, 14 migraine and 4 CH patients participated (mean duration 22.3 days). 133 headache attacks were registered (98 by migraine, 35 by CH patients). Strictly applying ICHD-3 criteria leads to 8/98 migraine without aura and 0/35 CH classifications. Adapted versions yield 28/98 migraine without aura and 17/35 CH classifications, with 12/18 participants having mostly diagnosis classifications when episodic TTH classifications (57/98 and 32/35) are ignored.

**Conclusions:**

Strictly applying the ICHD-3 criteria on individual attacks does not yield good classification results. Adapted versions yield better results, with the mostly classified phenotype (migraine without aura vs. CH) matching the diagnosis for 12/18 patients. The absolute number of migraine without aura and CH classifications is, however, rather low. Example cases can be identified where activity and stress events explain patient-reported headache triggers. Continuous improvement of the data collection protocol, ML algorithms, and headache classification criteria (including the investigation of integrating physiological data), will further improve future headache follow-up, classification and trigger detection.

*Trial registration* This trial was retrospectively registered with number NCT04949204 on 24 June 2021 at www.clinicaltrials.gov.

**Supplementary Information:**

The online version contains supplementary material available at 10.1186/s12911-022-01813-w.

## Background

### Introduction

Headache disorders are highly prevalent conditions and among the most disabling disorders globally [1]. In 2016, it was estimated by the World Health Organization (WHO) that approximately 1 in 2 adults had experienced a headache disorder at least once in the last year [2].

According to the International Classification of Headache Disorders, Third Edition (ICHD-3), headaches can be characterized as either primary or secondary [3]. Primary headaches are those for which the headache and its associated features are the disorder itself, while secondary headaches are caused by an underlying disorder [4]. The most common primary headache disorders are migraine, cluster headache (CH) and tension-type headache (TTH). Migraine is characterized by disabling headache attacks that last 4–72 h on average (if untreated) and are associated with symptoms, e.g., hypersensitivity to light and/or sound, nausea and/or vomiting. CH is characterized by shorter, severe and strictly unilateral headache attacks around the orbit or temple with associated symptoms such as restlessness and prominent ipsilateral cranial autonomic features. TTH, an almost universal experience for humans throughout life, was the third most prevalent condition worldwide in the Global Burden of Disease (GBD) study of 2016, with over one-fourth of the earth’s population having the occasional attack of TTH per year [5]. In addition to eliciting pain, headache disorders also affect different physiological systems such as the autonomic nervous system and the homeostatic mechanisms, leading to symptoms such as fatigue, gastro-intestinal alterations and hypersensitivity to stimuli. These symptoms may even be present hours to days before the actual headache attacks and bring additional disability to the patient [6, 7]. The discovery of these different phases (i.e., prodromal, aura, headache, postdromal) during headache attacks, highlights the fact that headache disorders are neurological disorders that encompass dynamic neurophysiological alterations both before and after headache attacks and thus not only during painful periods [6, 8]. In the absence of biological markers to reliably diagnose the different primary headache disorders, physicians rely on accurate classification of headache attacks to correctly diagnose patients, and to continuously follow up with them to optimize their therapy and health management [9, 10]. In current clinical practice, both diagnosis and follow-up happen intermittently during a doctor’s consultation through dialogue between patient and doctor. This means that physicians are dependent on intermittent subjective self-reporting by patients of historically experienced headache attacks and contextual factors (e.g., triggers).

The current gold standard for the diagnosis of headache disorders is the most recent ICHD-3, published by the International Headache Society (IHS) in 2018 [3]. The classification, established by headache experts, defines different diagnostic categories with specific criteria to classify a series of attacks as a certain disorder type, based on the information collected through dialogue between patient and physician (e.g., the nosological description and duration of several individual headache attacks) [11]. An ICHD-3 diagnosis is made when the attacks described by the patient match the criteria for an ICHD-3 defined disorder and when there is no better explanation for the symptomatology. Despite this, ICHD-3 only contains criteria for disorders and not for separate headache attacks as such for continuous follow-up. They are not designed and have not been field tested to classify individual headache attacks. In fact, today, no generic evidence-based system exists that is able to autonomously classify the type of an individual headache attack [12].

To follow up on headache attacks, paper diaries and different apps such as Migraine Buddy [13] are being used. Some apps offer features which try to move towards a more continuous follow-up outside a clinical setting but are still mainly dependent on self-reported information [14]. Hence, the physiological aspect is currently not taken into account during headache follow-up. In general, up to now, little to no exploratory research has been done to measure the physiological impact of having a primary headache disorder on a person’s lifestyle.

In the light of these opportunities and shortcomings associated with the current common practice, the exploratory mBrain study was started. Its main goal is to investigate how to move from the intermittent, subjective follow-up and classification of headache attacks and disorders based on self-reported data only, towards more continuous, prospective, semi-autonomous, multivariate and objective follow-up and classification, based on a combination of self-reported data and objective physiological and contextual data. mBrain is an observational and longitudinal study that focuses on patients diagnosed with episodic migraine with or without aura (ICHD-3 1.1 or 1.2), episodic cluster headache (ICHD-3 3.1.1), or chronic cluster headache (ICHD-3 3.1.2). During a trial period of approximately 3 weeks, participating patients are equipped with a wearable device and a smartphone that contains different applications used for data collection and follow-up. This way, the patient’s physiological and contextual data is collected from two sources: automatically via the wearable device and built-in smartphone sensors, and via a mobile application with a diary of the patient’s self-reported headache attacks. Using in-house designed machine learning (ML) algorithms, the automatically collected data is used to detect and recognize the patient’s physical activities (e.g. sitting, walking etc.), sleeping periods and stress periods. These predictions are shown in the custom developed mobile application and can either be confirmed or corrected by the patient.

The main research question of the mBrain study is whether its approach allows to generate new insights that can have a positive impact on the continuous follow-up and diagnosis of primary headache disorders. This way, the high amount of collected information about the patient-centered ecological context could allow for a better understanding of the impact of primary headache disorders on patient lives and vice versa. If the answer to this question is positive, mBrain might be valuable to both patients and doctors in several ways, for example by improving diagnosis through the help of an automatic headache classification system, the personalization of treatment plans, or the prediction of future headache attacks.

### International Classification of Headache Disorders, 3rd edition

As a basis for the development of an autonomous headache classification system, the obvious starting point is the set of ICHD-3 criteria, since it is the current standard for diagnosing headache disorders. However, the fact that the ICHD-3 criteria are designed to be used by a physician during consultations, raises the question whether they can be applied as classification criteria in a continuous follow-up setting outside the walls of the physician’s office, as is intended during the mBrain study. In addition, since they are designed to diagnose a disorder based on multiple headache attacks, it should be researched whether criteria for the classification of individual headache attacks can be extracted.

ICHD-3 already mentions the benefit of keeping a headache diary in which important characteristics of headache attacks are recorded. Apart from being helpful in teaching patients to differentiate between different headache types themselves, it has been shown that this improves the accuracy of clinical diagnoses [15, 16].

### Related work

Today, different digital tools exist that can be used by headache patients to follow up on their headache syndromes [14]. Most of them are commercially available smartphone applications that solely focus on migraine [14]. The most well-known, most downloaded and highest rated application in this area is Migraine Buddy [13]. Key features are customizable attack recording and automatic sleep detection purely based on smartphone data. Besides sleep and weather, the app is based on only self-reported information. Other applications focusing on migraine include iMigraine [17], Migraine Headache Diary HeadApp [18] and Migraine Monitor [19]. Some applications focus on specific other primary headache disorders, such as My Cluster Headache [20] and Tension Headache [21]. Some general logging applications exist, such as Headache Log [22].

In research, headache apps have helped patients to control their acute medication usage. As overuse may lead to chronification of headaches, this may help the outcome of medication withdrawal in patients with medication-overuse headaches [23]. In recent years, apps are also being tested to provide behavioral therapy and telemedicine for headache disorders [24].

In general, studies have shown that self-reporting apps can be effective tools towards the improvement of self-managing headache disorders, and the mediation of the interaction between headache patients and their doctors [25, 26]. However, the evidence base to show their effectiveness and clinical safety is not strong, and psychometrics are almost never taken into account [24, 26, 27]. Therefore, the inclusion of electronic devices such as wearables offers interesting additional options for the follow-up of headaches [24, 28]. Doing all of this, the privacy of patients should never be exposed, which is still an issue in many commercial apps [29]. Other criteria that are often forgotten about but are also important, include the usability of the app and personalization features [14].

In addition, digital tools exist that focus on the autonomous classification and diagnosis of headache attacks [30]. Importantly, previous studies recommend combining clinical interviews and a diagnostic diary for the follow-up and diagnosis of headache disorders, and to never purely rely on an autonomous system [31]. Nevertheless, there is still a need for tools that combine both aspects, to support clinicians in the classification and diagnosis [30]. Especially for the classification of *individual* headache attacks, no evidence-based research has sufficiently investigated this path [12]. One study specifically examined the classification of individual attacks as either migraine or TTH [12], by slightly adapting the ICHD-3 diagnostic criteria. However, the classification algorithm was not designed to discern any other headache disorder types.

The emerging potential of healthcare technology has been recognized by the IHS. Recently, the Clinical Trials Subcommittee published a position paper on the implementation of health technology assessments in clinical trials for medications or medical devices for the acute and preventive treatment of migraine. They recognize the importance of new technologies for the collection and analysis of evidence for the treatment of migraine and to facilitate health technology assessments that account for the distinctive nature of migraine and the heterogeneity of the affected population [32]. This statement was published after the start of this research project and start of this trial.

### Paper objective and organization

Several subquestions of the main research question need to be investigated:

- How can a system be set up that collects objective, explicit data about a patient’s headache attacks and relevant context?
- How should a system be designed that autonomously classifies individual headache attacks? What criteria can be used for this classification, based on the available collected data? Can the ICHD-3 criteria be used for this purpose?
- Can physiological wearable data give an accurate view on contextual information such as the patient’s activities, stress periods, and sleeping behavior?
- Is it actually useful to objectively map the context of headache attacks experienced by patients with migraine and CH? How can this physiological, contextual and headache-related data be linked to be valuable for the continuous follow-up and/or classification of headaches?

The objective of this paper is to investigate these individual research questions to assess the potential benefit of the continuous follow-up of headache patients using a combination of objective and self-reported data. First, the paper describes all details of the mBrain data collection set-up. Spread over two data collection waves, a total of 18 migraine or CH patients have already participated in the study. By analyzing various statistics and the impact of changes made between both waves, research questions (a) and (c) are reviewed. Second, the paper proposes the design of a preliminary, autonomous, knowledge-based classification system for individual headache attacks, starting from ICHD-3. It is evaluated on the available data from headache attack registrations by the study participants, to answer research question (b), and to further identify the requirements needed to improve its design. Finally, the paper investigates research question (d), by analyzing whether and how contextual information of a patient can be used in a knowledge-based trigger detection system and early warning system for headache events.

## Methods

To let migraine and CH patients participate in the mBrain study, a data collection protocol was designed and deployed. This section covers all aspects of this set-up: the wearable, the data-driven ML algorithms, the mobile applications, the data collection protocol, and the technical system architecture. Moreover, it discusses the design of a preliminary version of a knowledge-based headache classification system for individual headache attacks, and a possible methodology for the knowledge-based detection of predefined headache triggers.

### Wearable: the Empatica E4

The wearable used in the mBrain study to collect the participant’s physiological data, is the Empatica E4 [33]. This is a CE-certified medical-grade wearable device that offers physiological data acquisition in real-time. It has an internal memory that can store up to 36 h of data, but also offers the option to send the data in real-time over a Bluetooth Low Energy (BLE) connection to a smartphone. The Empatica E4 consists of different physiological sensors:

- a photoplethysmogram (PPG) sensor, which measures blood volume pulse (BVP) (64 Hz frequency), from which heart rate (HR) and the inter beat interval of the heart rate (IBI) can be derived
- a 3-axis accelerometer (32 Hz frequency)
- an electrodermal activity (EDA) sensor, which measures the galvanic skin response (GSR) (4 Hz frequency)
- an infrared thermopile, which measures skin temperature (4 Hz frequency)

In the mBrain study, the BLE streaming mode of the Empatica E4 is used.

### Data-driven machine learning algorithms

To get insight in the activities, sleeping behavior, and perceived stress of a participant, different data-driven ML algorithms were designed. Their goal is to accurately predict these events from the preprocessed objective physiological data collected by the Empatica E4 wearable. Four different algorithms were designed:

- Activity recognition: this algorithm determines whether a person is sitting, standing, lying down, walking, running, or cycling. It computes statistical features of the accelerometer signal only on a rolling window of 15 s with 50% overlap, which are then fed to a catboost (gradient boosted trees) model every 7.5 s. Smoothing is used to correct (obvious) mispredictions and arrive to minute-level predictions. In the final step, these 1-min predictions are aggregated per 5-min interval according to a fixed set of rules.
- Commute detection: this algorithm determines whether a person is commuting, based on the collected location data and the output of the activity recognition algorithm. For every predicted activity, it calculates the movement speed based on the time and distance covered between the first and last location sample in the prediction interval. If the moving speed is higher than 25 km/h, the predicted activity type is corrected to commuting.
- Sleep detection: this algorithm determines the person’s time-to-bed, get-up time, sleep duration and preliminary sleep quality measures taking into account the number of wake-up periods between the time-to-bed and get-up timestamps. The algorithm analyzes the values of the activity index as described by Cole et al., averaged over windows of 5 min, that exceed predefined thresholds [34]. Aggregated windows above the threshold shorter than 1 h, defined within a large period of rather low activity index values, are reported as wake-up periods. Larger aggregated windows above the threshold are used to determine the time-to-bed and get-up time. As a post-processing step, an anomaly detection algorithm analyzes for which sleeping periods the measured sleep quality is significantly lower than normal.
- Stress detection: this algorithm predicts the probability on a minute-wise granularity that a person is experiencing acute stress versus no stress. Using wearable data, it measures the physiological component of the stress-response, which is characterized by sympathetic nervous system (SNS) activations [35]. Note that not-stress related events, such as exercise, can also influence SNS activations. The algorithm makes use of the E4’s skin conductance and temperature signals and is trained on the publicly available WESAD dataset [36].

More details on the activity recognition, commute detection and sleep detection algorithms can be found in Steenwinckel et al. [37].

### Mobile applications

Three mobile applications were installed on the participant’s smartphone. Two Android applications were designed for this study: mBrain and Empatica Streamer. The third app, OwnTracks [38], is an external application used for location tracking.

During the first data collection wave of the mBrain study, feedback of participants on the mBrain application was collected. This feedback, together with some observations made by the researchers, was used to improve the mBrain v1 baseline application of the first wave, to mBrain v2 used for the second wave.

#### mBrain v1

**Fig. 1:**
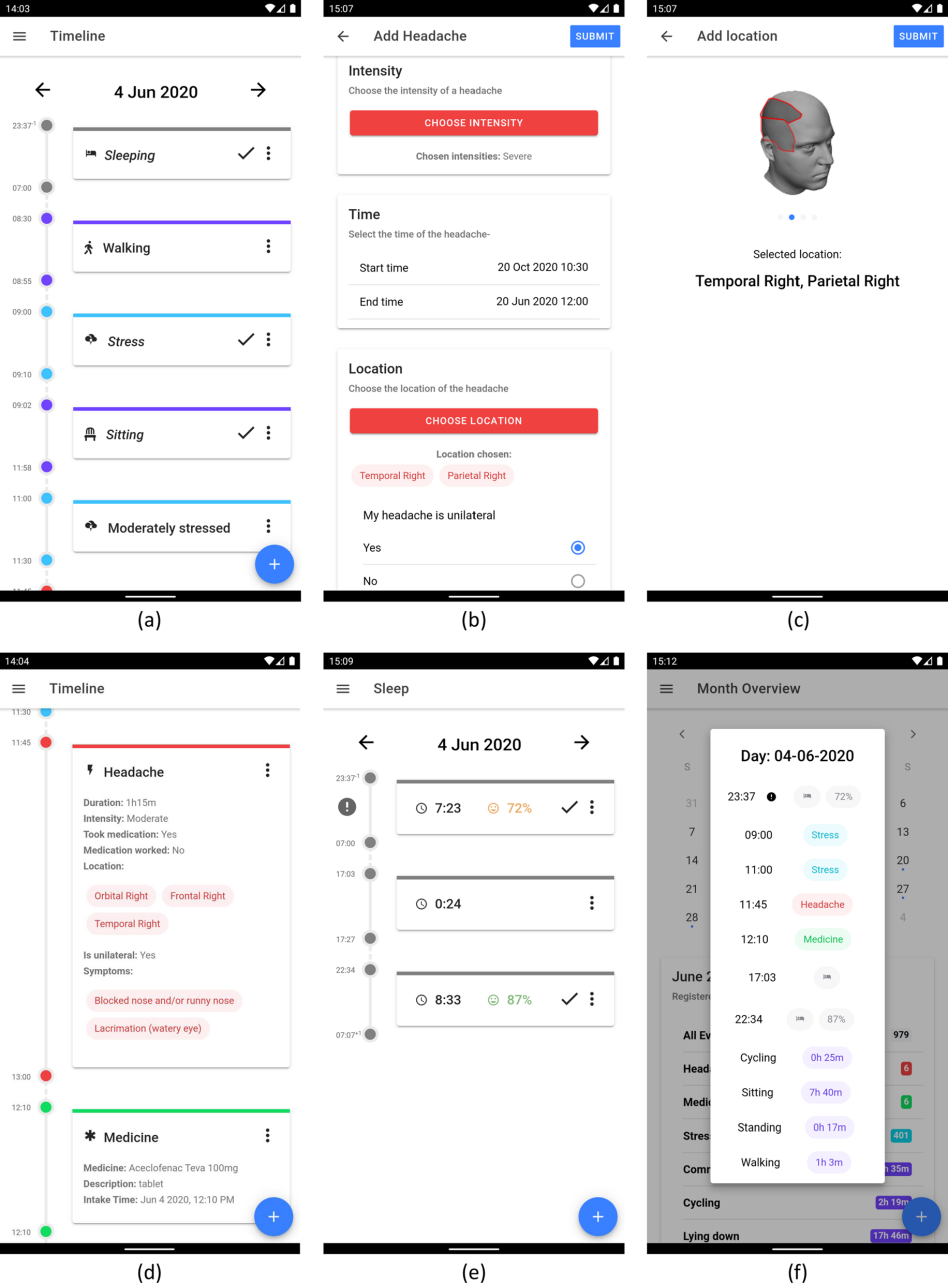
Screenshots of the mBrain v1 application. (**a**) Timeline with events; (**b**) Register headache attack; (**c**) Select headache location; (**d**) Timeline with new headache attack; (**e**) Sleep overview; (**f**) Month overview with daily summaries

The mBrain application is used by the participants to keep track of all relevant contextual data about their daily life. Screenshots of mBrain v1 are shown in Fig. 1.

##### Account set-up

Each participant of the mBrain study can set up an account in the mBrain app, in cooperation with the accompanying physician-researcher, and receives a unique patient ID. This patient ID is used to identify all collected data of this participant. Moreover, it is used by the accompanying physician-researcher to link the account to the concrete participating patient, which, for obvious privacy reasons, is unknown to the other non-medical researchers involved in the mBrain study. During set-up, the participant also selects his or her personal acute medications for headaches (e.g., analgesics such as acetaminophen or disorder-specific drugs such as triptans, ergotamines or oxygen therapy for CH).

##### Event registration

The mBrain application allows the participant to keep track of different types of events: headache attacks, activities of daily living, sleeping periods, stress periods, medicine intakes, and (if applicable) menstrual periods. Tables 1 and 2 detail the information that is requested for the registration of the different event types. In practice, participants register their headache attacks and medicine intakes themselves, and interact with activity, stress and sleep events that are automatically added to their timeline of events. Figure 1b and c show some screenshots of the registration of a headache attack.

**Table 1:** Information requested in the mBrain app for the registration of the different event types

Event type	Requested information
Stress	Start time; end time; stress intensity (no stress (0), moderate stress (1), high stress (2))
Activity	Start time; end time; activity type (sedentary, sitting, standing, lying down, walking, running, cycling, commuting, other [any type of activity is allowed, of which a textual description required])
Sleep	Time to bed; wake-up time
Medicine intake	Time; medicine name, dose and form
Headache attack	Start time; end time; pain intensity (Table 2); headache location(s) (Table 2); pain being unilateral (yes, no); headache symptom(s)* (Table 2); headache trigger(s)* (Table 2); acute medication intake (yes and successful, yes but unsuccessful, no)
Period (if applicable)	Start time; end time

For items with a predefined set of options, the options are mentioned between brackets, or the table with the options is referred to between brackets. In mBrain v1, all information except information with an asterisk (*) is required. In mBrain v2, all information is required

**Table 2:** Requested input and available options when registering a headache attack in the mBrain app

Requested information	Available options
Pain intensity	No pain (0), mild pain (1), moderate pain (2), severe pain (3), very severe pain (4)
Headache location(s)	Cervical left; cervical mid; cervical right; frontal left; frontal mid; frontal right; mandibular left; mandibular right; maxillar left; maxillar right; occipital left; occipital mid; occipital right; orbital left; orbital right; parietal left; parietal mid; parietal right; temporal left; temporal right
Headache symptom(s)	Conjunctival injection; lacrimation; ptosis; miosis; eyelid oedema; nasal congestion; rhinorrhoea; sweaty forehead and face; pulsating pain; movement sensitivity / pain increment during routine physical activity; restlessness or agitation; photophobia; phonophobia; osmophobia; nausea; vomiting
Headache trigger(s)	Alcohol; atmospheric pressure difference; bright light; caffeine; change in weather; cold; coughing; decreased water intake; flickering light; heat; height; holiday; illnesses; loud sounds; medication; menstrual cycle; physical exercise; pressing; relieve from stress; resolvents; sexual intercourse; skipping of meals; sleep deprivation; sleeping away; smells/odors; sneezing; specific head movements; stress; touch

The information is applicable to both mBrain v1 and v2. For all information except pain intensity, more than one option can be selected by the participant. In mBrain v2, the option “none of those” can also be selected for headache symptoms and headache triggers if no other option is selected. The available options for pain intensity and headache symptoms are based on the diagnostic criteria of migraine and cluster headache in ICHD-3 [3]

For the registration of headache attacks, an ICHD-3 based approach was developed by the physician-researchers of the mBrain study team to define the terminology used for pain severity, pain location and headache symptomatology. For severity, a five-point Likert scale approach was used in accordance with intensity levels of ICHD-3 (no pain, mild pain, moderate pain, severe pain and very-severe pain). For headache location, an interactive manikin with topographical anatomical landmarks was developed, where participants can register one or many zones of pain during a headache attack. The ICHD-3 terminology on headache associated symptoms was translated into layman’s terms in Dutch by the physician-researchers in accordance with common clinical terminology in headache medicine, since no formal ICHD-3 translation in Dutch existed at the time of the study.

##### Timeline of events

The timeline shows a chronological overview of all events in the selected day. It consists of events registered by the participant, and events automatically added by the ML algorithms. Overlapping events are shown sequentially. Figure 1a and d show some screenshots of the timeline.

For the events that are automatically added by the data-driven ML algorithms, the participant is asked to interact with them *as much as possible* to let the system know whether these events are correct or not. In this way, the correctness of the ML algorithms can be validated, which is important for further improvement and allows to shift towards personalization of the algorithms. If an event is correct, the participant can easily confirm the event by hitting a check mark button. If an event is incorrect, the participant can edit or remove it.

##### Sleep overview

Specifically for sleep events, the participant is able to view a dedicated sleep overview per day. Additional information is shown for automatically added sleep events, based on the output of the sleep ML algorithm: an estimation of the quality of the sleep (percentage) and a visual indication when this quality is significantly lower than normal (via an exclamation mark). An example of this sleep overview is visualized in Fig. 1e.

##### Daily records

The participant is requested to fill in and submit daily reports with the following information: the general stress level during the day (on a scale from 1 to 10, 10 meaning the highest possible stress level); the general mood during the day (on a scale from 1 to 5, 5 meaning the best possible mood); whether or not each of the three main meals (breakfast, lunch, dinner) has been taken, and the time of consumption for taken meals. The participant can fill this in at any time.

##### Other functionality

The participant can hit the event mark button on a connected Empatica E4 to register specific moments in time. These timestamps are saved and shown on the tag page for later use, e.g., to easily register a headache attack or edit an event predicted by the ML algorithms. Moreover, the month overview shows statistics about all events in the timeline, aggregated per month as well as summarized per day. A screenshot of this is shown in Fig. 1f.

#### mBrain v2

Based on observations by the researchers and feedback collected from participants of the first data collection wave with mBrain v1, mBrain v2 was created for usage during the second wave. This section highlights the changes of mBrain v2 compared to mBrain v1. A motivation for these changes and a discussion of their impact, is given in the “Discussion” section.

##### Symptom and trigger input required for headache registration

In mBrain v2, the requirement is added that a participant needs to select at least one symptom and trigger when registering a headache attack. To accommodate the situation where no symptom or trigger is applicable, the new option “none of those” can be selected.

##### Timeline changes

The following things have changed in the timeline of mBrain v2:

- The timeline is split up in two views: a normal view and a detailed view. The detailed view shows the exact same timeline as in mBrain v1. The normal view is the new view that abstracts sedentary activities. A sedentary activity is an activity with type “sedentary”, “sitting”, “standing” or “lying down”. All sedentary activities are labeled as “sedentary”, and grouped when they follow up on each other in time (i.e., when there are at most 60 s in between), and when they are either all confirmed or all unconfirmed. This way, the number of individual events in the timeline is significantly reduced, since sedentary events take up the largest part of the detailed timeline. Interactions with sedentary events in the normal timeline view are reflected in the other timeline view. A confirmation of a sedentary event in the normal view only means that the event is confirmed as being of sedentary form, but not explicitly as *sitting*, *standing* or *lying down* if that is the predicted label, unless the individual event is also confirmed in the detailed timeline view. As especially dynamic behavior impacts migraine [39], this grouping of sedentary events has no negative impact on migraine management, but highly reduces the required feedback of participants: it allows the participant to give coarse-grained feedback whenever giving fine-grained feedback is not feasible. The normal view is the default view when opening the timeline page.
- The number of stress events that are automatically added to the participant’s timeline by the stress detection ML algorithm is limited to at most 2 events per hour and at most 10 events per day. Within a single execution of the stress detection algorithm, the newly predicted stress periods are sorted from longest to shortest duration. Next, this list is processed in order, where each event is only added to the timeline if the applicable hourly and daily limits both have not yet been reached.
- Whenever a stress event is added to the timeline that ended not longer than 15 min ago, a mobile notification is sent to the smartphone of the participant.
- For every automatically added sedentary activity in the timeline, the participant’s location at the start time and end time of the activity is compared. If there is a significant difference between both locations, the activity is flagged and a visual exclamation sign is added to this event in the timeline, indicating a potential misprediction to the participant.

**Fig. 2:**
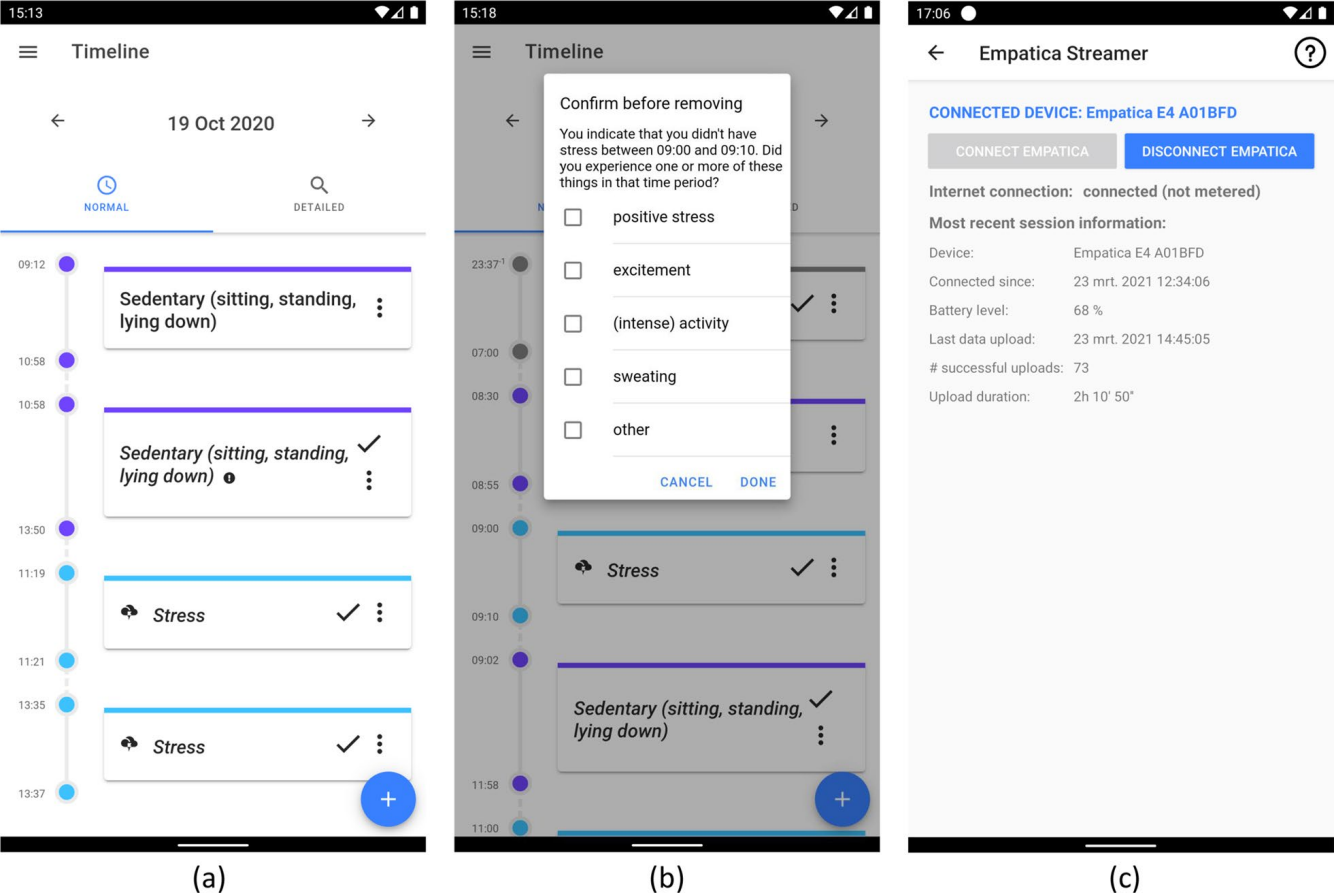
Screenshots of the mBrain v2 and Empatica Streamer applications. (**a**) mBrain v2: normal and detailed timeline view; (**b**) mBrain v2: remove predicted stress event; (**c**) Empatica Streamer: connected with an Empatica E4

In Fig. 2a, a screenshot of the updated timeline of mBrain v2 is shown.

##### Additional input requested when confirming or deleting a predicted stress event

When the participant removes an incorrect automatically added stress event from the timeline in mBrain v2, he or she is asked to specify what was experienced during the time of the mispredicted period. At least one of the following options should be selected: positive stress; excitement; (intense) movement or activity; sweating; other (none of the available options). A screenshot of this new input request is shown in Fig. 2b. Moreover, the participant is explicitly requested to select the intensity (moderate stress or high stress) when confirming an automatically added stress event, as no stress level is predicted by the ML algorithm.

#### Empatica Streamer

The Empatica Streamer application is a separate application installed on the participant’s smartphone, which can only be opened from the menu of the mBrain app. It allows the participant to connect an Empatica E4 device to the smartphone via BLE. Once connected, the Empatica E4 will stream the physiological data in real-time to the smartphone, which buffers the data and chronologically uploads it over WiFi to the server environment. Via the application and permanent notifications, the participant can keep track of the connection status. Figure 2c shows a screenshot of the main page of the Empatica Streamer app while it has an active BLE connection with an Empatica E4 device.

#### OwnTracks

OwnTracks [38] is an external application that is used to collect the participant’s location data. For the mBrain study, OwnTracks is configured to upload the smartphone’s GPS coordinates and an estimated accuracy every *X* minutes, provided that there is at least a 50 m difference to the previously uploaded coordinates. During the first data collection wave, *X* was set to 3 min. For the second wave, *X* was updated to 0.5 min, to allow for better route reconstruction for high velocity movements such as driving or cycling.

### Protocol of data collection trial

The data collection trial with actual headache patients was performed in cooperation with physician-researchers from the Department of Neurology of the Ghent University Hospital. The protocol has been approved by the Ethics Committee of the Ghent University Hospital (BC-07403). The methods in the protocol are in accordance with all relevant guidelines and regulations.

#### Inclusion and exclusion criteria

To participate in the mBrain study, the inclusion criteria were defined as follows:

1. The patient is an adult between 18 and 65 years old.
2. Only one of the following two criteria is fulfilled:
  - The patient is diagnosed with *migraine with aura* or *migraine without aura*. The diagnosis is made based on the diagnostic criteria 1.1 or 1.2 of ICHD-3 [3], respectively. The diagnosis exists for at least 1 year.
  - The patient is diagnosed with *cluster headache*. The diagnosis is made based on the diagnostic criteria 3.1 of ICHD-3 [3]. The diagnosis exists for at least 1 year.

The following exclusion criteria were defined:

1. The patient is diagnosed with any other headache disorder (other than the ones specified by inclusion criterion 2) that makes the classification of a headache attack more difficult, with the exception of comorbid infrequent or frequent episodic TTH (ICHD-3 2.1 or 2.2) if those episodes are clearly distinguishable from attacks of migraine or CH [40].
2. The patient is suffering or has recently suffered from alcohol and/or drug abuse.
3. The patient has significant medical comorbidity that can cause interference with the research, according to the judgment of the physician-researcher.
4. The patient is traveling to a foreign country during the trial period.
5. The patient is using beta blockers.
6. The patient is participating in any other academic or commercial clinical trial.

##### Additional criteria for migraine patients

For patients fulfilling inclusion criterion 2a, the following additional inclusion criteria were defined:

1. The patient has had less than 15 days with headache per month during the past 3 months.
2. The patient has at least 2 migraine attacks per month.
3. Migraine attacks started when the patient was younger than 50 years.
4. The patient’s migraine attacks can be clearly distinguished from any other headache disorder.

##### Additional criteria for CH patients

For patients fulfilling inclusion criterion 2b, the following additional inclusion criteria were defined:

1. CH attacks started when the patient was younger than 50 years.
2. The patient expects to have at least 5 CH attacks per week.
3. The patient’s CH can be clearly distinguished from any other headache disorder.

Only patients with an Android smartphone could participate in the study. However, different Android smartphones were available for patients without an Android smartphone such that they could still participate, if all other criteria were fulfilled.

#### Patient recruitment and start-of-trial intake visit

Patients eligible to participate were recruited via the headache outpatient clinic and communication channels of the Department of Neurology of the Ghent University Hospital. The intake and outtake visits took place at Ghent University Hospital.

During the intake visit, the participant received detailed information from the physician-researcher about the goals of the study, the rights of the participants, and the study safety procedures. The participant was requested to read the information letter of the study and sign an Informed Consent Agreement. Next, the physician-researcher took a detailed history on baseline demographic characteristics, headache-related current and previous medication usage and headache characteristics. Thereafter, the physician asked the participant to fill in multiple questionnaires:

- The Migraine Disability Assessment Test (MIDAS), Dutch version [41–43]
- The MOS Short-Form General Health Survey (SF-20) [44, 45]
- Migraine-Specific Quality-of-Life Questionnaire (MSQ Version 2.1) [46] (only for patients diagnosed with *migraine with aura* or *migraine without aura*)

After this, the Empatica E4 was given to the participant. The three mobile applications were installed on the participant’s smartphone. If the participant did not possess an Android smartphone, he or she was given a smartphone with the applications preinstalled. The physician-researcher set up the different applications together with the participant. Finally, the participant received a user manual with detailed instructions and guidelines about the trial and the different mobile applications. The most important instructions were also explained and demonstrated by the physician-researcher. Subjects did not receive any compensation for participating in the study apart from a parking ticket voucher.

#### Continuous follow-up during trial period

The trial period of each participant took 21 days, with a possible deviation of a few days depending on the participant’s personal agenda. During this period, the participant was requested to adhere to the following guidelines:

- The operating system specific and application-specific settings of the installed applications must not be changed, except for the in-app settings of the mBrain application. Bluetooth should be enabled at all times to retain connection with the Empatica E4 and WiFi should be enabled as much as possible to enable the uploading of the collected data.
- The Empatica E4 wearable should be worn as much as possible. During these periods, the Empatica E4 should be connected to the smartphone via the Empatica Streamer application as much as possible. During an active connection, the Empatica E4 and the smartphone used for the trial should be kept as close as possible to each other, to avoid a Bluetooth disconnection which causes the Empatica data streaming to stop until a manual reconnection. To avoid unintentional disconnections, the connection status should be regularly checked via the Empatica Streamer application and the Empatica device itself. Since the battery of the Empatica E4 lasts approximately 6–12 h in streaming mode, the Empatica must be charged at least two times per day, but not during nighttime whilst sleeping. Instead, the preferred schedule is to charge the Empatica in the evening, wear a fully charged Empatica at least 1 h before going to bed, and keep wearing the Empatica while sleeping.
- A correct and detailed diary of all events should be kept via the mBrain application. This request is threefold. First, automatically added events (activities, sleep periods and stress periods) in the event timeline should be interacted with as much as possible by either confirming, editing or removing them. Second, all other relevant events that are not automatically added to the timeline should be registered. This involves headache attacks, acute medicine intakes and menstrual periods (if applicable), as well as activities, sleep periods and stress periods that are not automatically added to the timeline. Third, the daily record should be filled in every day. For all information logged in the mBrain app, and especially for the registration of headache attacks, it is important to be as precise and complete as possible.

These guidelines were communicated to the participant during the trial intake visit and were also listed in the user manual given to the participant.

#### End-of-trial outtake visit

When the participant’s trial period ended, he or she had a final outtake visit with the physician-researcher. During this visit, all used devices were returned. In preparation of the outtake visit, the participant’s collected data was observed and analyzed by mBrain’s researchers. Based on this, a report was created that the physician-researcher discussed with the participant during the outtake visit. This report contained questions to help resolving any missing or incomplete data observed during the analysis, questions to clarify interesting observations made by the researchers, and questions related to the interaction with and general feeling about the mobile applications. This process allowed to get new insights and improve further iterations of the applications and ML algorithms.

### Technical architecture

**Fig. 3:**
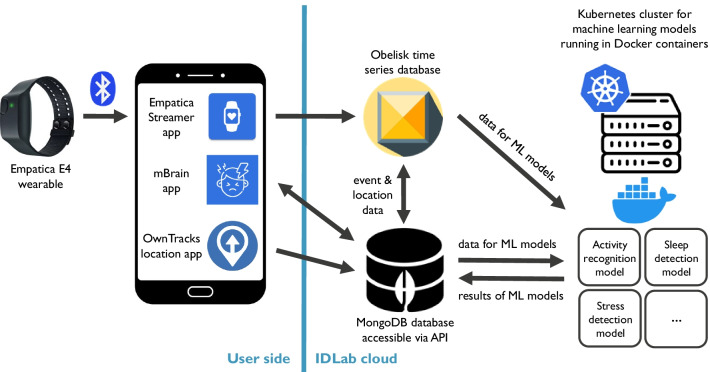
Architectural set-up of the mBrain data collection system

The architecture of the mBrain data collection system is shown in Fig. 3. It contains the user side and the server environment hosted on the IDLab cloud.

On the user side, the three mobile applications (mBrain, Empatica Streamer and OwnTracks) are installed on an Android smartphone. The Empatica Streamer app makes a connection with the Empatica E4, which streams its measured physiological data over a BLE connection to the smartphone. The applications communicate with the server environment over a secured HTTPS connection.

The server environment is hosted on the IDLab cloud, which is the research group’s in-house cloud environment. For mBrain, it hosts three main parts. First, it hosts Obelisk, which is an existing platform for building scalable applications using time-series data [47]. Obelisk is used for storing all collected high-frequency Empatica data, except for the output of the PPG sensor, which was not stored due to technical constraints. This data is sent to Obelisk from the Empatica Streamer app for ingestion, and is available for querying to the other server components. Second, the server environment contains a MongoDB [48] database instance which stores all other mBrain-related data. It is accessible via the mBrain application programming interface (API). This API is used by the mBrain app for communicating to the server environment, and by OwnTracks to upload the patient’s location data. Third, a Kubernetes [49] cluster is deployed on the iLab.t Virtual Wall [50] portion of the server environment. This cluster is used to automatically and reliably schedule and execute individual runs of the different ML algorithms in Docker containers [51]. The activity recognition, commute detection and stress detection algorithms are executed every 5 min, while the sleep detection algorithm runs every 24 h. These algorithms each query the relevant Empatica data from Obelisk for the processed time period, and fetch other relevant data from the MongoDB database via the mBrain API. Using this data, the algorithms run their predictive models and send the automatically generated events to the mBrain API.

### Knowledge-based classification of individual headache attacks

This section proposes the design of a preliminary, autonomous, i.e., system-based, knowledge-based classification system for individual headache attacks, based on the data collected in the mBrain study, starting from the ICHD-3 diagnostic criteria.

#### Usage of semantics and design of mBrain ontology

For the design of the classification system, a semantic approach is applied. This enables the consolidation of the available data, as it imposes a common, machine-interpretable data representation [52]. To do so, the mBrain ontology has been designed. This ontology is a semantic model that formally describes the mBrain domain knowledge through its concepts and their relationships and attributes [53]. It allows to semantically describe all details of a headache attack and a patient’s contextual information, to be used for the knowledge-based classification of headache attacks and detection of headache triggers.

For the headache-specific aspects, the mBrain ontology contains the classification hierarchy of the different headache disorders. For the primary headache disorders, the hierarchy is worked out more completely in terms of subcategories specified in ICHD-3. A semantic classification system needs to start from classifying an individual headache attack and could then potentially use these individual classifications to assess a person’s general disorder diagnosis. Hence, the ontology makes a distinction between an individual headache attack that can be of a certain headache phenotype, and the general diagnosis of a patient. Moreover, the ontology should at least contain all concepts related to the ICHD-3 diagnostic criteria of the primary headache disorders focused on in the mBrain project, i.e., migraine, CH, and frequent/infrequent episodic TTH. Nevertheless, its generic design allows to easily extend the ontology for other headache disorder types and phenotypes in the future.

The mBrain ontology is built as an extension of the DAHCC (Data Analytics for Health and Connected Care) ontology [54]. This general ontology is internally designed to describe everything related to the collection of raw sensor data, contextual data and ML predictions. This linking is important to enable hybrid AI, where the data-driven ML outputs can be used in knowledge-based systems. The DAHCC ontology is a combination of multiple, existing ontologies, enriched with information and concepts specific for its purpose. These ontologies include SAREF4EHAW (the Smart Applications REFerence ontology extended with concepts of the eHealth Ageing Well domain) [55] and SSN (Semantic Sensor Network) [56].

**Fig. 4:**
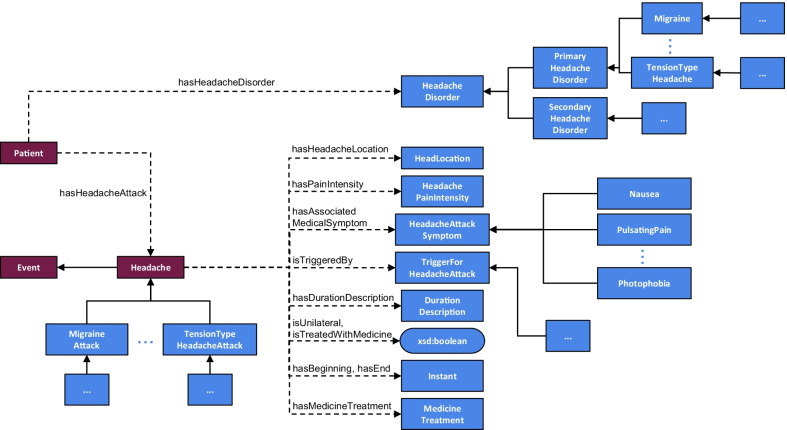
Overview of the most important concepts in the mBrain ontology, and the relations between them. The blue concepts are the new concepts introduced in the mBrain ontology, while the red concepts are the concepts that exist in the DAHCC ontology, with which the mBrain ontology links

Figure 4 shows a high-level overview of the most important concepts in the mBrain ontology, the relations between them, and the link with the DAHCC ontology. In Listing 1, a semantic representation is given of a headache attack, predicted activity and predicted stress event with the mBrain ontology.
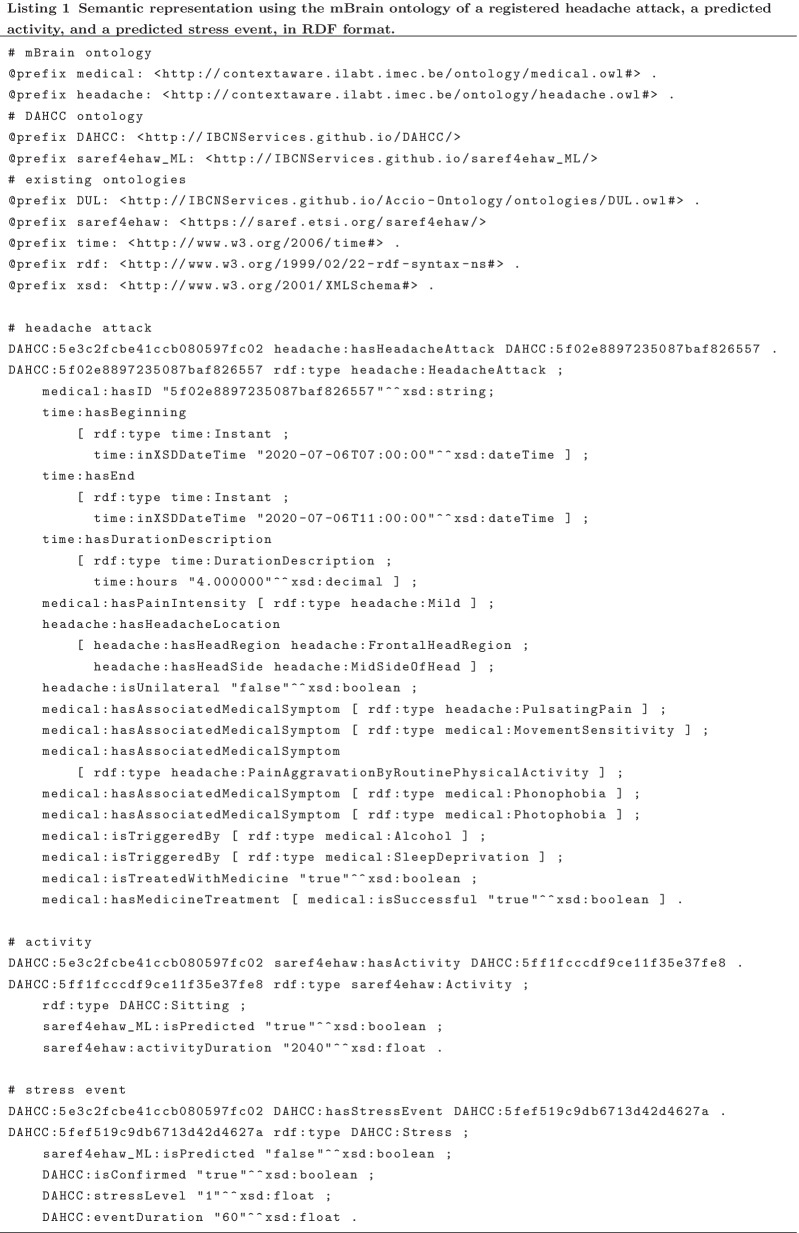

#### Requirements of the classification system

The inclusion criteria of the mBrain study allow patients diagnosed with *migraine without aura*, *migraine with aura*, and *CH* to participate in the study. Moreover, since TTH is the most common primary headache disorder [2, 3], it cannot be ignored. Hence, this classification system will focus on migraine, CH and TTH.

For the design of a knowledge-based classification system, ICHD-3 is chosen as the starting point. The focus of our research is on the headache attacks. We included patients with migraine with aura, however, we did not specifically investigate aura as a separate phenomenon. Therefore, in the headache classification system, the focus will be on migraine following the criteria of migraine without aura (ICHD-3 1.1). For TTH phenotype, we take note that ICHD-3 makes a further distinction between *infrequent* and *frequent* episodic TTH. However, the diagnostic criteria of both disorders only differ in the frequency of individual episodes, while the diagnostic criteria applicable to individual episodes are identical. Therefore, the classification for individual headache episodes only differentiates between TTH or not. Note that the term infrequent or frequent does hence not matter for the type of the individual episode and will therefore be omitted for the remainder of this paper. Other attack phenotypes such as thunderclap headache or stabbing/paroxysmal headaches, are currently out of the scope of our research.

#### Classification criteria

Three versions of classification criteria for individual headache attacks were designed in chronological order. The motivation for each new version follows from generating and analyzing the headache attack registrations and their classification results. This motivation will be further elaborated on in the “Discussion” section. For the remainder of this paper, note that the term “classification criteria” always refers to the criteria applied by the designed classification system, as opposed to the term “diagnostic criteria” which refers to the ICHD-3 criteria.

##### Version 1 of the classification criteria

The first version of the classification criteria consists of exactly these ICHD-3 criteria that are targeted at individual attacks. To obtain them, all criteria that do not relate to an individual attack were put in a separate set (a). This set includes criteria targeted at the frequency, total number and/or time period of the attacks, as well as the caution criterion “Not better accounted for by another ICHD-3 diagnosis” which is a reminder to always consider other diagnoses that might better explain the headache. These criteria in set (a) can be ignored for the classification of an individual headache attack.

##### Version 2 of the classification criteria

To properly assess the headache duration and characteristics, the ICHD-3 diagnostic criteria for *migraine without aura* and *CH* require the headache attack to be “untreated or unsuccessfully treated”, and “untreated”, respectively. As a consequence, (successfully) treated attacks are not taken into account during clinical diagnosis based on ICHD-3. Hence, they do not meet version 1 of the classification criteria. However, in practice, both migraine and CH patients treat their attacks with medication, and often with success. Therefore, in version 2, all classification criteria of version 1 are again applied strictly, except for the criteria related to medication treatment. Concretely, this means that for *migraine without aura*, it is no longer required that the attack is “untreated or unsuccessfully treated”. Similarly, it is not required that an attack is “untreated” to be classified as a *CH* attack.

##### Version 3 of the classification criteria

In close collaboration with headache experts, the following decisions were made in a 3rd version of the classification criteria:

- The ICHD-3 criteria for *migraine without aura* and *CH* state a required duration, which is conditioned on the attack being “untreated or unsuccessfully treated”, and “untreated”, respectively. In version 2, the treatment conditions for both disorders are no longer required. However, (successfull) treatment may have an influence on the perceived duration of the attack and symptomatology. In fact, ICHD-3 does not define the required duration of a (successfully) treated attack. Hence, for version 3 of the criteria, the condition on the specified duration of an attack does also not need to be fulfilled.
- For *CH*, the ICHD-3 criteria state that during less than 50% of the active time course of a cluster period with attacks, attacks may be less severe and/or of shorter or longer duration, as compared to the diagnostic criteria. This is another reason to ignore the duration criterion in version 3 of the classification criteria. Moreover, specifically for the classification of *CH* attacks, it also leads to the decision to not require the fulfillment of the severity criterion.

##### Overview of the different versions of the classification criteria

Table 3 shows an overview of how the ICHD-3 diagnostic criteria are mapped to the classification criteria of *migraine without aura*, *CH* and *episodic TTH*. For each disorder type, the diagnostic criteria are divided in three different disjunctive sets:

- the set of criteria that are not targeted at characteristics of individual attacks—they are ignored by all versions of the classification criteria for individual headache attacks;
- the set of criteria targeted at characteristics of individual attacks, that *need* to be fulfilled *by version 3 of the classification criteria*;
- and the remaining set of criteria targeted at characteristics of individual attacks, that do *not* need to be fulfilled *by version 3 of the classification criteria*.

In the remainder of this paper, parts of the criteria in set (c) are often referred to as the *treatment criterion*, *duration criterion* and *severity criterion*. Table 4 makes explicit which parts of the criteria are exactly meant by those terms.

**Table 3:** Constructed sets of classification criteria based on ICHD-3 for the headache classification system

Disorder type	Set	Criteria
Migraine without aura	Set (a)	A: At least five attacks fulfilling criteria B–D
		E: Not better accounted for by another ICHD-3 diagnosis
	Set (b)	C: Headache has at least two of the following four characteristics:
		(1) unilateral location
		(2) pulsating quality
		(3) moderate or severe pain intensity
		(4) aggravation by or causing avoidance of routine physical activity
		D: During headache at least one of the following:
		(1) nausea and/or vomiting
		(2) photophobia and phonophobia
	Set (c)	B: Headache attacks lasting 4–72 h (when untreated or unsuccessfully treated)
Cluster headache	Set (a)	A: At least five attacks fulfilling criteria B–D
		D: Occurring with a frequency between one every other day and eight per day
		E: Not better accounted for by another ICHD-3 diagnosis
	Set (b)	B (part): Unilateral orbital, supra-orbital and/or temporal pain
		C: Either or both of the following:
		(1) At least one of the following symptoms or signs, ipsilateral to the headache:
		(a) Conjunctival injection and/or lacrimation
		(b) Nasal congestion and/or rhinorrhoea
		(c) Eyelid oedema
		(d) Forehead and facial sweating
		(e) Miosis and/or ptosis
		(2) A sense of restlessness or agitation
	Set (c)	B (part): Severe or very severe pain lasting 15–180 min (when untreated)
Episodic tension-type headache	Set (a)	A: At least 10 episodes of headache occurring on < 1 day/month on average (< 12 days/year) and fulfilling criteria B–D
		E: Not better accounted for by another ICHD-3 diagnosis
	Set (b)	C: At least two of the following four characteristics:
		(1) Bilateral location
		(2) Pressing or tightening (non-pulsating) quality
		(3) Mild or moderate intensity
		(4) Not aggravated by routine physical activity
		D: Both of the following:
		(1) No nausea or vomiting
		(2) No more than one of photophobia or phonophobia
	Set (c)	B: Lasting from 30 min to 7 days

The table gives an overview of how the ICHD-3 criteria of *migraine without aura* (criteria 1.1), *cluster headache* (criteria 3.1) and *episodic tension-type headache* (criteria 2.1 for *infrequent* episodic tension-type headache), are split up in three sets for version 3 of the classification criteria for an individual headache attack: set (a) with criteria not targeted at individual attacks; set (b) with criteria targeted at individual attacks that are required to classify an attack as this type; and set (c) with criteria targeted at individual attacks that are *not* required to classify an attack as this type. The mentioned letters refer to the criteria letters as how they are presented in ICHD-3. Note that for version 1 of the classification criteria, both set (b) and set (c) are required for the classification of an individual attack as that type. For version 2, all criteria in set (b) and set (c) are required for classification, except for the treatment criteria (see Tables 4 and 5)

In summary, Table 5 shows the concrete criteria that are required for the classification of individual headache attacks as the different types, in the different versions of the classification criteria. In addition, it also highlights for each version of the criteria, which individual criteria are additionally evaluated for each headache attack that is classified as that type. This will be further explained in the next section. To make the overview in Table 5 clear, the table uses the names of the sets mentioned in Table 3, and the criterion names made explicit in Table 4.

**Table 4:** Mapping of criterion types to ICHD-3 criteria in set (c) of Table 3

Disorder type	Duration criterion	Treatment criterion	Severity criterion
Migraine without aura	Attack lasting 4–72 h	Attack untreated or unsuccessfully treated	/
Cluster headache	Pain lasting 15–180 min	Attack untreated	Severe or very severe pain
Episodic tension-type headache	Pain lasting 30 min to 7 days	/	/

The table gives an overview of how the ICHD-3 criteria in set (c) (i.e., the criteria targeted at individual attacks that are *not* required to classify an attack as the corresponding type *in version 3* of the classification criteria, see Table 3) of the different disorder types are mapped onto the different criterion types: *duration*, *treatment* and *severity*. If set (c) does not contain the criterion type, the table cell is empty (“/”)

**Table 5:** Required and additionally evaluated criteria for all versions of classification criteria for individual headache attacks

	Required criteria	Additionally evaluated criteria
Version 1	Set (b) and Set (c)	/
Version 2	Set (b) and Set (c), excluding treatment criterion	Treatment criterion
Version 3	Set (b)	Duration, treatment and severity criterion

The table presents which ICHD-3 diagnostic criteria are used as actual *required* classification criteria in the different versions of the classification criteria for individual headache attacks of the different disorder types, and which criteria are additionally evaluated to enrich the classification output. To make this overview compact and clear, the names of the sets from Table 3 and criterion names from Table 4 are used

#### Methodology of the classification system

This section presents the methodology of the semantic classification system for individual headache attacks.

##### Classification input

The input of the classification system consists of the semantic description of a headache attack registered via the mBrain app with the mBrain ontology, of which an example is presented in Listing 1.

##### Classification output

The classification criteria are validated for each individual headache attack, independently from other events and independently per type. Hence, it is checked separately for each of the three disorder types whether the attack can be classified as that type. This means that the output of classifying an individual headache attack is a set of zero to three individual classifications.

One classification contains two things: (i) the type of disorder the attack is classified as, and (ii) a binary indication of fulfillment for each criterion that is targeted at an individual headache attack but not required for the classification, according to the considered version of the classification criteria. The criteria in this set are specified as “additionally evaluated criteria” in Table 5. The rationale behind this is that it is still interesting for a person (e.g., a physician) to know whether the unrequired criteria are actually fulfilled or not; this gives more information than a classified type only, and can be important for treatment.

##### Classification process

The classification criteria of each disorder type have been translated into a semantic query that is executed on a data store containing the mBrain ontology data and the semantic representation of the headache attack that needs to be classified. Moreover, the additionally evaluated criteria have also been translated into a set of additional simple queries, which need to be executed after the main classification query. Altogether, this results in one set of queries per considered headache disorder phenotype, which need to be executed in order.

In terms of technologies, all semantic data is represented in Resource Description Framework (RDF) format [57]. The SPARQL Protocol and RDF Query Language (SPARQL) is used to write and evaluate the queries [58]. To manage the query execution process, including the addition and deletion of events to the data store and the semantic reasoning, any semantic reasoning engine such as Apache Jena or RDFox [59] can be used.

In Listing 2, an example of how the version 3 classification criteria are translated into the main SPARQL classification query is given for *migraine without aura*. 
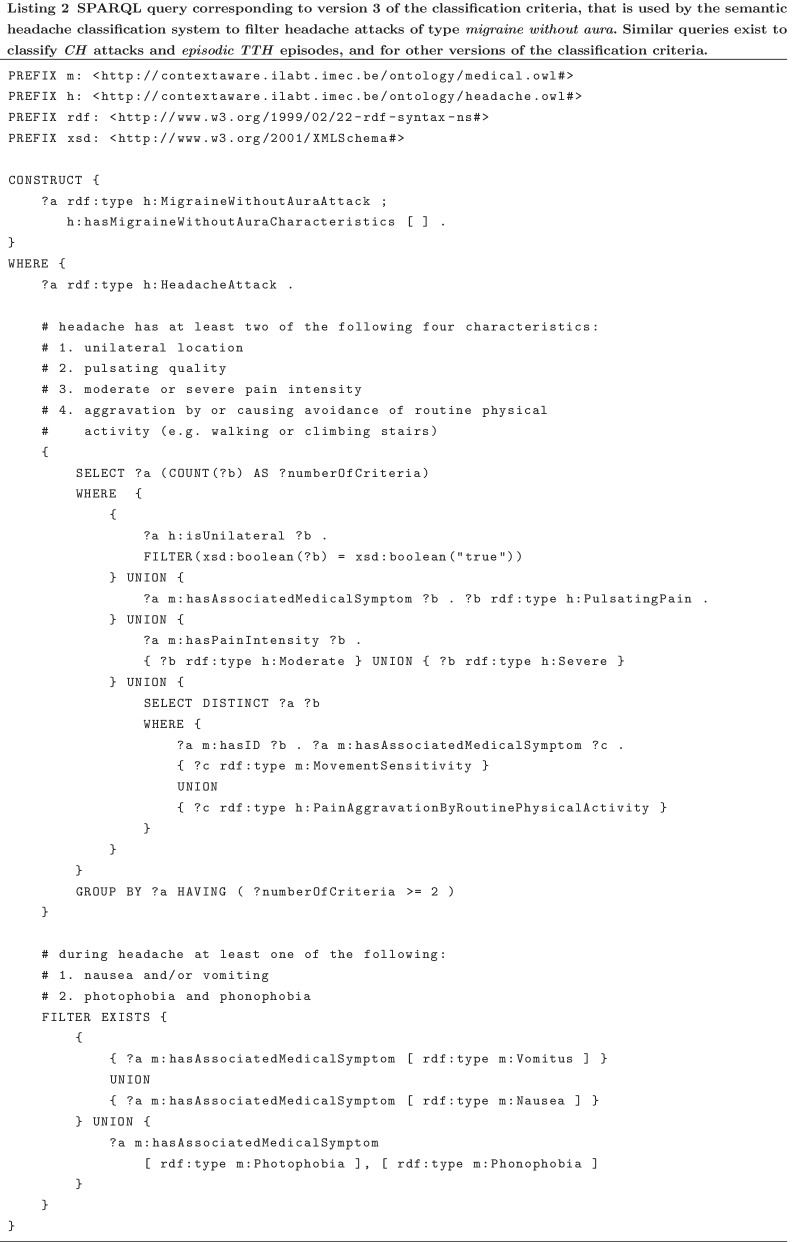


#### Knowledge-based diagnosis of headache disorders

Based on the classification of individual headache events experienced by a patient, a second step can be to also classify them as a specific disorder, as this is semantically equivalent to diagnosing that patient with a specific disorder. To do so, the same semantic classification system can be applied, with a set of disorder classification queries. The criteria that should be used for this in a first version, are those that are not targeted at individual headache attacks but specify the frequency and time period of the attacks that fulfill the other ICHD-3 criteria. This corresponds to the set of criteria that were omitted for the classification of individual headache attacks, i.e., the criteria in set (a) of each disorder type in Table 3.

### Knowledge-based detection of headache triggers

This section discusses the design of a preliminary knowledge-based trigger detection system evaluating triggers for headache at a personal level. In headache medicine, certain triggers are well-known (e.g., menstrual cycle in women with migraine, alcohol in CH patients), but others are debated. The question often remains whether certain events, behaviors or external factors can truly be classified as triggering the attack, or as premonitory (prodromal) phenomena of the attack. In clinical practice, physicians and even patients find it hard to disentangle this question and leave the suggestion that certain symptoms may be misattributed by patients as triggers, but basically are headache associated symptoms at the start of the attack [60, 61].

A preliminary knowledge-based trigger detection system could—given the correct knowledge about triggers and an accurate detection of them using the collected data—potentially be a valuable tool for patients with regular headache attacks. Such a system takes advantage of the wide range of contextual data collected in the mBrain study. In the mBrain app, upon the registration of a headache attack, patients can select any possible triggers for that attack out of a list. This list, which is specified in Table 2, originates from medical expert knowledge on headache attacks, and especially migraine and CH [62, 63]. For each trigger in this list, the question is whether the occurrence of this trigger can be detected based on the data collected in the mBrain study. Based on the automatically generated activity, stress and sleep events and the collected contextual data, the occurrence of five headache triggers out of the provided trigger list could potentially be detected: physical exercise, sleep deprivation, stress, relieve from stress, and skipping of meals.

For a semantic trigger detection system to actually detect the occurrence of any of these events/situations, queries should be written that can be automatically executed on a data window of specified duration. This window should contain all physiological and contextual data and events of interest that are collected and generated within its boundaries. This is where the mBrain ontology again has an important role: it provides a means to semantically describe and link all this data in a common, machine-interpretable format. The size of the data window also needs to be defined dynamically based on medical expert knowledge, taking into account information about patient and context, as this makes the assumption that the trigger lies within this time range. If a query detects that a known trigger for a patient has occurred, it could for example generate an alarm that a headache attack might follow for that patient, which could be translated into a mobile notification of the mBrain app.

In Listing 3, a very simple illustrative example is given of a stream processing query that could detect the physical exercise trigger. It checks whether physical exercise is a known trigger for any existing patient in the data store, and if so, whether any activity representing physical exercise has been detected for this patient with a duration of at least 5 min in the considered time window. If this is the case, a headache alarm is generated.
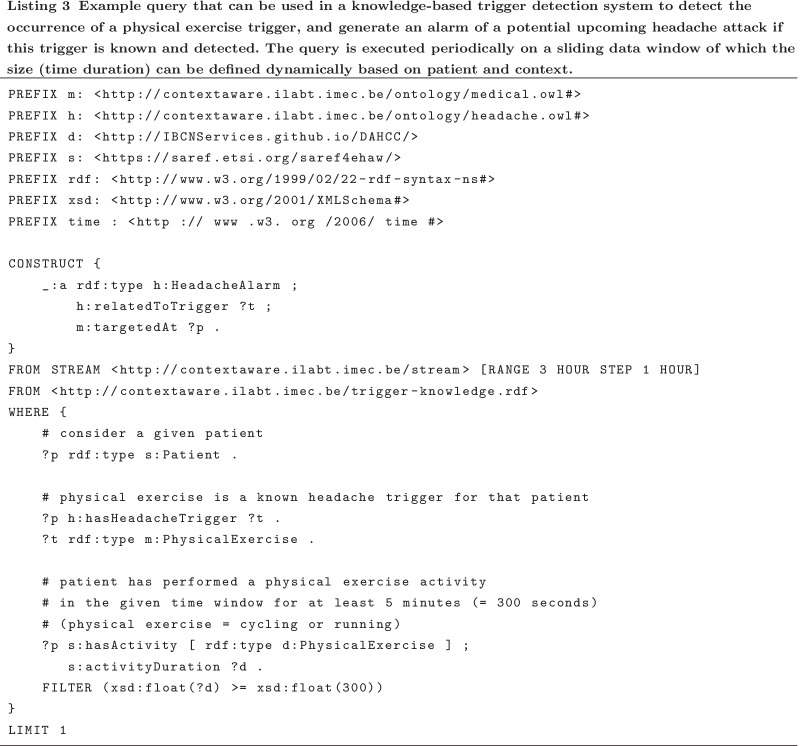


## Results

During the period from July 2020 until October 2020, 7 patients participated in the first data collection wave of the mBrain study: 5 migraine patients and 2 CH patients. In the second data collection wave, which took place from October 2020 until January 2021, 11 more patients participated: 9 migraine patients and 2 CH patients. First wave participants used the mBrain v1 application, while second wave participants used mBrain v2. All CH patients were chronic CH patients (ICHD-3 3.1.2). This section presents the first results of the mBrain study.

### Data collection results

Table 6 describes the general demographic characteristics of the study subjects. Table 7 presents their current and previous acute and preventive medication use.

**Table 6:** Demographics and baseline characteristics of the group of mBrain participants

	Total group	Migraine	Cluster headache
	(n = 18)	(n = 14)	(n = 4)
Age (years), mean (SD)	39.1 (11.9)	37.1 (10.6)	46 (15.4)
Sex (female), pct. (ratio)	66.7% (12/18)	85.7% (12/14)	0% (0/4)
Migraine days per month, mean (SD)	/	5.2 (2.2)	/
Days with headache attacks per month, mean (SD)	/	/	23.8 (7.5)
Weight (kg), mean (SD)	74.3 (13.5)	70.0 (11.1)	89.2 (10.4)
Height (cm), mean (SD)	173 (8.5)	170 (6.9)	182 (5.6)
# children, median (IQR)	1 (0–2)	1 (0–2)	1 (0–1)
Alcoholic beverages per week (units), mean (SD)	2.3 (2.9)	1.7 (1.9)	4.3 (5.1)
Cigarettes per day (units), median (IQR)	0 (0–2)	0 (0–0)	9 (6–11)

Numbers are presented for the total group, as well as separately for the migraine and cluster headache patients in the participant group. The results are provided for wave 1 and wave 2 combined. Abbreviations used in this table: pct. is percentage, SD is standard deviation, IQR is interquartile range, “#” represents “number of”

**Table 7:** Current and previous use of medications for headache disorder in the group of mBrain participants

	Total group	Migraine	Cluster headache
	(n = 18)	(n = 14)	(n = 4)
Current use of acute medication, pct.	100%	100%	100%
Current # acute medications in use, median (IQR)	3 (2–3)	3 (2–3)	3 (2–4)
Current use of preventive medication, pct.	88.9%	85.7%	100%
Current # preventive medications in use, median (IQR)	1 (1–2)	1 (1–1)	1 (1–1)
# previous acute medications used, median (IQR)	2 (1–3)	2 (1–4)	0 (0–1)
# previous preventive medications used, median (IQR)	2 (1–4)	1 (1–4)	1 (1–3)

Numbers are presented for the total group, as well as separately for the migraine and cluster headache patients in the participant group. The results are provided for wave 1 and wave 2 combined. Abbreviations used in this table: pct. is percentage, IQR is interquartile range, “#” represents “number of”

Table 8 shows the general statistics of the first and second data collection waves. It zooms in on the data collection, timeline activity and interaction, and daily records. An additional file shows the same statistics complemented by spread measures where appropriate, in two separate tables (one per wave) (see Additional file 1).

**Table 8:** General statistics of data collection during the first and second mBrain data collection wave

	Wave 1	Wave 2
# patients	7	11
Duration of trial (days) (pp), mean	22.57	22.09
Data collection statistics		
Connected Empatica time per trial day (HH:mm) (pp), mean	09:06	12:49
# location points per trial day (pp), mean	91.49	109.78
# tags per trial day (pp), mean	0.41	0.89
Timeline activity and interaction statistics		
Headache attacks		
# headache attacks (pp), mean	5.71	8.45
Pct. of patients w/o any headache attack	14.29%	0.00%
Medicine intakes		
# medicine intakes (pp), mean	3.86	5.64
Pct. of patients w/o any medicine intake	57.14%	9.09%
Activities		
# man. activities (pp), mean	1.07	2.60
# aut. activities per trial day (pp), mean	35.81	46.24
Pct. of aut. activities of sedentary type (pp), mean	90.42%	88.29%
Pct. of aut. activities *fully* confirmed (pp), mean	19.19%	45.90%
Pct. of aut. activities *only* confirmed as sedentary, but w/o explicit confirmation of predicted type (pp), mean	0.00%	12.01%
Pct. of aut. activities w/ *only* corrected time (pp), mean	0.04%	0.56%
Pct. of aut. activities w/ corrected type (pp), mean	6.34%	5.31%
Pct. of aut. activities removed (pp), mean	3.22%	10.27%
Pct. of aut. activities ignored (pp), mean	71.20%	25.94%
Sleep periods		
# man. sleep pds. (pp), mean	13.86	19.45
# aut. sleep pds. (pp), mean	31.57	37.36
Pct. of aut. sleep pds. confirmed (pp), mean	6.11%	13.46%
Pct. of aut. sleep pds. w/ corrected time (pp), mean	0.48%	2.62%
Pct. of aut. sleep pds. corrected to activity (pp), mean	0.00%	0.70%
Pct. of aut. sleep pds. removed (pp), mean	5.46%	23.43%
Pct. of aut. sleep pds. ignored (pp), mean	87.96%	59.79%
Stress periods		
# man. stress pds. per trial day (pp), mean	0.04	0.05
# aut. stress pds. per trial day (pp), mean	15.86	6.66
Pct. of aut. stress pds. confirmed w/o level (pp), mean	2.63%	0.00%
Pct. of aut. stress pds. confirmed w/ level 1/2 (pp), mean	1.48%	44.89%
Pct. of aut. stress pds. corrected w/ level 0 (pp), mean	17.49%	28.62%
Pct. of aut. stress pds. removed (pp), mean	5.00%	2.66%
Pct. of aut. stress pds. w/ corrected time (pp), mean	0.03%	0.04%
Pct. of aut. stress pds. ignored (pp), mean	73.37%	23.79%
Daily record (DR) statistics		
Pct. of trial days w/ part of DR provided (pp), mean	78.89%	92.99%
Pct. of trial days w/ daily stress level provided (pp), mean	73.07%	91.34%
Pct. of trial days w/ daily mood provided (pp), mean	73.66%	91.34%
Pct. of trial days w/ daily food intake provided (pp), mean	60.17%	87.62%
Pct. of trial days w/ DR fully completed (pp), mean	58.87%	87.62%

Statistics mentioning “(pp)” are “per patient” statistics: they are first calculated per patient, and then aggregated over all patients. Other abbreviations used in this table: aut. is automatically added, man. is manually added, pct. is percentage, pds. is periods, w/ is with, w/o is without, “#” represents “number of”. An additional file shows two separate tables (one per wave) highlighting the same statistics complemented by spread measures where appropriate (see Additional file 1)

In Table 9, statistics are shown about the headache attacks registered during the two data collection waves, split up based on the diagnosis of the participants. They compare the intensity, duration, location, symptoms and triggers of the attacks.

**Table 9:** Statistics of registered headache attacks during the first and second mBrain data collection wave

	Migraine		Cluster headache	
	W1	W2	W1	W2
# patients	5	9	2	2
Duration of trial (days), mean (SD)	22.60	22.11	22.50	22.00
	(1.95)	(0.33)	(0.71)	(0.00)
Total # headache attacks	20	78	20	15
# attacks per patient, mean (SD)	4.00	8.67	10.00	7.50
	(2.74)	(3.46)	(7.07)	(0.71)
Pain intensity of attacks, mean (SD)	2.00	1.76	1.50	2.27
	(0.92)	(0.81)	(0.61)	(0.70)
Pct. of attacks w/ pain intensity				
0 (no pain)	0.00%	0.00%	0.00%	0.00%
1 (mild pain)	30.00%	46.15%	55.00%	13.33%
2 (moderate pain)	50.00%	33.33%	40.00%	46.67%
3 (severe pain)	10.00%	19.23%	5.00%	40.00%
4 (very severe pain)	10.00%	1.28%	0.00%	0.00%
Duration (HH:mm), mean (SD)	08:27	05:59	00:22	00:50
	(09:39)	(05:42)	(00:15)	(00:20)
Pct. of unilateral attacks	45.00%	73.08%	100.00%	100.00%
# symptoms per attack, mean (SD)	4.40	2.29	0.65	2.73
	(2.41)	(2.10)	(0.67)	(1.03)
# triggers per attack, mean (SD)	0.75	0.83	0.35	0.33
	(0.79)	(0.90)	(0.49)	(0.49)
Pct. of attacks w/o symptom	0.00%	19.23%	45.00%	0.00%
Pct. of attacks w/o trigger	45.00%	43.59%	65.00%	66.67%
Pct. of attacks treated	95.00%	51.28%	70.00%	100.00%
Pct. of attacks treated successfully	75.00%	37.18%	70.00%	93.33%
Pct. of attacks w/ selected symptom:				
Conjunctival injection	10.00%	0.00%	5.00%	26.67%
Lacrimation	15.00%	5.13%	0.00%	26.67%
Ptosis	0.00%	8.97%	0.00%	0.00%
Miosis	0.00%	6.41%	0.00%	0.00%
Eyelid oedema	5.00%	6.41%	10.00%	0.00%
Nasal congestion	20.00%	7.69%	0.00%	80.00%
Rhinorrhoea	20.00%	3.85%	0.00%	6.67%
Sweaty forehead and face	25.00%	1.28%	0.00%	0.00%
Pulsating pain	75.00%	34.62%	45.00%	53.33%
Movement sensitivity	40.00%	28.21%	0.00%	6.67%
Pain increment during routine physical activity	40.00%	25.64%	0.00%	6.67%
Restlessness or agitation	30.00%	21.79%	5.00%	60.00%
Photophobia	95.00%	24.36%	0.00%	6.67%
Phonophobia	35.00%	29.49%	0.00%	0.00%
Osmophobia	20.00%	2.56%	0.00%	0.00%
Nausea	10.00%	23.08%	0.00%	0.00%
Vomiting	0.00%	0.00%	0.00%	0.00%

The statistics are split up based on the diagnosis of the participating patients. Columns entitled W1 and W2 present the results for wave 1 and wave 2, respectively. Abbreviations used in this table: pct. is percentage, SD is standard deviation, w/ is with, w/o is without, “#” represents “number of”

### Knowledge-based headache classification results

The proposed knowledge-based classification system for headache attacks, presented in the “Knowledge-based classification of individual headache attacks” section, has been applied on the headache data collected during the two data collection waves.

Table 10 focuses on the migraine patients and shows the number of classifications of the attacks experienced by the migraine patients as *migraine without aura*, *CH* and *episodic TTH*, using the three versions of the classification criteria. It shows the results for both the first data collection wave (n = 20 attacks) and the second wave (n = 78 attacks). A similar overview is shown in Table 11 for the CH patients in the first wave (n = 20 attacks) and second wave (n = 15 attacks).

**Table 10:** Results of applying the classification criteria on registered headache attacks of the migraine participants

	Criteria v1		Criteria v2		Criteria v3	
Classification	W1	W2	W1	W2	W1	W2
Migraine without aura	1	7	5	13	7	21
	(5.00%)	(8.97%)	(25.00%)	(16.67%)	(35.00%)	(26.92%)
CH	0	0	0	0	4	15
	(0.00%)	(0.00%)	(0.00%)	(0.00%)	(20.00%)	(19.23%)
Episodic TTH	10	45	10	45	11	46
	(50.00%)	(57.69%)	(50.00%)	(57.69%)	(55.00%)	(58.97%)

These results are the output of applying the different versions of the classification criteria for individual headache attacks on the headache attacks experienced by the 14 migraine patients that have successfully participated in the first and second wave of the mBrain study (5 in wave 1, 9 in wave 2). Columns entitled W1 and W2 present the results for wave 1 and wave 2, respectively. The numbers in the cells represent the number of attacks that are classified as the type specified in the row header, out of the total number of attacks registered by migraine patients during that wave (i.e., 20 attacks for wave 1, 78 attacks for wave 2); the percentages of these ratios are given between brackets. Abbreviations used in this table: CH is cluster headache, TTH is tension-type headache

**Table 11:** Results of applying the classification criteria on registered headache attacks of the cluster headache participants

	Criteria v1		Criteria v2		Criteria v3	
Classification	W1	W2	W1	W2	W1	W2
Migraine without aura	0	0	0	0	0	0
	(0.00%)	(0.00%)	(0.00%)	(0.00%)	(0.00%)	(0.00%)
CH	0	0	0	6	3	14
	(0.00%)	(0.00%)	(0.00%)	(40.00%)	(15.00%)	(93.33%)
Episodic TTH	2	12	2	12	19	13
	(10.00%)	(80.00%)	(10.00%)	(80.00%)	(95.00%)	(86.67%)

These results are the output of applying the different versions of the classification criteria for individual headache attacks on the headache attacks experienced by the 4 CH patients that have successfully participated in the first and second wave of the mBrain study (2 in wave 1, 2 in wave 2). Columns entitled W1 and W2 present the results for wave 1 and wave 2, respectively. The numbers in the cells represent the number of attacks that are classified as the type specified in the row header, out of the total number of attacks registered by CH patients during that wave (i.e., 20 attacks for wave 1, 15 attacks for wave 2); the percentages of these ratios are given between brackets. Abbreviations used in this table: CH is cluster headache, TTH is tension-type headache

Table 12 further details the results of applying version 3 of the classification criteria on the registered headache attacks. It also shows how often the additionally evaluated criteria are fulfilled for the classifications of each type. Moreover, it makes an analysis of whether the diagnosis of the migraine and CH patients corresponds to the disorder type for which the highest number of headache attacks is classified as that type, as compared to the other types. We refer to this as patients with “mostly diagnosis classifications”.

**Table 12:** Results of applying version 3 of the classification criteria on all registered headache attacks

	Migraine		Cluster headache	
	W1	W2	W1	W2
# patients	5	9	2	2
Total # headache attacks	20	78	20	15
# patients with mostly diagnosis classif.	2	2	0	2
(pct. of # patients)	(40.00%)	(22.22%)	(0.00%)	(100.00%)
# patients with mostly diagnosis classif.,				
Not considering *episodic TTH* classif.	3	6	1	2
(pct. of # patients)	(60.00%)	(66.67%)	(50.00%)	(100.00%)
Classif. of attacks as *migraine without aura*				
# classif. (pct. of # headache attacks)	7	21	0	0
	(35.00%)	(26.92%)	(0.00%)	(0.00%)
# classif. with fulfilled duration criterion	5	13	0	0
# classif. with fulfilled treatment criterion	1	9	0	0
# classif. with all criteria fulfilled	1	7	0	0
# registered symptoms per	6.86	4.57		
classification, mean (SD)	(1.77)	(2.40)	/	/
Classif. of attacks as *CH*				
# classif. (pct. of # headache attacks)	4	15	3	14
	(20.00%)	(19.23%)	(15.00%)	(93.33%)
# classif. with fulfilled duration criterion	2	3	3	14
# classif. with fulfilled treatment criterion	0	5	0	0
# classif. with fulfilled severity criterion	0	5	0	6
# classif. with all criteria fulfilled	0	0	0	0
# registered symptoms per	7.50	4.07	1.67	2.86
classification, mean (SD)	(1.29)	(2.81)	(0.58)	(0.95)
Classif. of attacks as *episodic TTH* episodes				
# classif. (pct. of # headache attacks)	11	46	19	13
	(55.00%)	(58.97%)	(95.00%)	(86.67%)
# classif. with fulfilled duration criterion	10	45	2	12
# registered symptoms per	2.73	1.57	0.63	2.54
classification, mean (SD)	(1.27)	(0.81)	(0.68)	(0.88)
# classif. per attack				
# attacks with 0 classif.	1	9	1	0
(pct. of # headache attacks)	(5.00%)	(11.54%)	(5.00%)	(0.00%)
# attacks with 1 classif.	16	56	16	3
(pct. of # headache attacks)	(80.00%)	(71.79%)	(80.00%)	(20.00%)
# attacks with 2 classif.	3	13	3	12
(pct. of # headache attacks)	(15.00%)	(16.67%)	(15.00%)	(80.00%)
# attacks with 3 classif.	0	0	0	0
(pct. of # headache attacks)	(0.00%)	(0.00%)	(0.00%)	(0.00%)

The table presents the results of applying version 3 of the classification criteria for individual headache attacks on the 133 headache attacks experienced by the 18 patients that have successfully participated in the first and second data collection wave of the mBrain study, split up based on the diagnosis of the participating patients. Columns entitled W1 and W2 present the results for wave 1 and wave 2, respectively. Patients with mostly diagnosis classifications are patients for who the patient’s diagnosis matches the disorder type for which there are the most classifications out of the patient’s headache attacks. Abbreviations used in this table: classif. is classifications, CH is cluster headache, TTH is tension-type headache, pct. is percentage, SD is standard deviation, “#” represents “number of”

### Knowledge-based trigger detection results

A knowledge-based headache trigger detection system, following the methodology presented in the “Knowledge-based detection of headache triggers” section, offers the tools to detect the occurrence of some known triggers based on the data collected in the mBrain study. Currently, the only source of knowledge about headache attack triggers is the set of triggers indicated by the patient himself. As a first step to investigate the potential of a knowledge-based trigger detection system, this section checks with two example cases whether indicated, detectable triggers can actually be backed up with the collected physiological data, i.e., whether one can find proof in the data that the trigger actually occurred in the period preceding the headache attack. In other words, this is equivalent to investigating whether a query-based system would have been able to detect the trigger for the attack.

The first example case involves the “physical exercise” trigger. In a simple set-up (see Listing 3), this trigger could be detected by checking if the patient has performed any activity representing physical exercise in the considered time window. Given the activity types detectable by the ML algorithms, this includes any activity of type running or cycling (excluding commuting, since our definition of commuting requires no real physical exercise). Out of all mBrain patients, 2 migraine patients have indicated “physical exercise” as a trigger for a total of 4 headache attacks. For 2 headache attacks, a running event has actually taken place in the period of 3 h before the attack. The details of one example are given below:
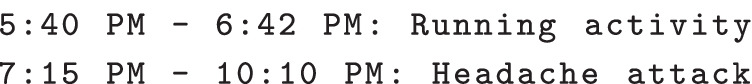


A second example case investigates the “stress” trigger. Stress as a trigger requires more knowledge about the type and period of stress, to write an accurate query to detect it. Nevertheless, the number of stress events is evaluated for the 11 headache attacks with stress as indicated trigger, experienced by 3 migraine patients. For 6 of the 11 attacks, at least one confirmed stress event was observed in the period of 5 h before the attack. For 4 of those, this was also the case in the hour preceding the attack. When combining multiple stress events and observing their total duration in a certain period, longer periods of stress become visible preceding some attacks for one specific patient. A good example to illustrate this is the following:
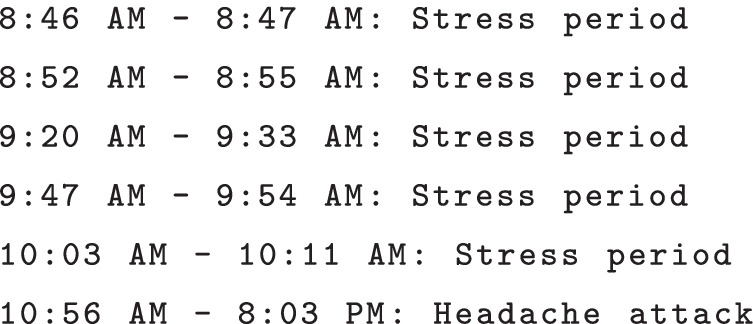


## Discussion

Based on the results of the first and second data collection wave of the mBrain study presented in the “Results’’ section, several aspects can be discussed with respect to the objectives of this paper outlined in the “Paper objective and organization” section.

### Knowledge-based classification of individual headache attacks

#### Headache registrations

As can be observed in Table 9, 98 headache attacks have been registered by the migraine patients that participated in the mBrain study, and 35 attacks by the CH patients. This leads to a total of 133 registered attacks.

Looking at the attacks of the first data collection wave, a first observation was the fact that sometimes, no symptoms and/or triggers were selected by the patient. This information was not required in mBrain v1. In separate headache events, it is possible that a patient does not experience any of the symptoms in the list, or believes no item in the trigger list was a probable trigger for the attack. Patients with migraine or CH may also experience episodes of TTH, which is characterized by the absence of certain migraine or CH specific symptoms (see the ICHD-3 criteria for TTH, Table 3). In the analysis of the headache attacks, all unselected information is implicitly assumed to be not applicable. In mBrain wave 1, this assumes that patients process all available symptoms and triggers, and *only* select none, *if* none are applicable. It is important that this assumption is true, especially related to the selection of the symptoms, since those variables are used as input for the headache classification. However, from the collected participant feedback, it appeared that some patients simply did not always check (part of) the list of symptoms and triggers, because of lack of interest or time. Hence, in mBrain v2, the requirement was added that a patient needs to select at least one symptom and trigger when registering a headache attack, with the option at the bottom to select “none of those”, which then functions as the new ground truth entry for absence of headache-associated symptomatology or trigger for the particular attack.

Surprisingly, the results on the headache attacks registered during the second wave, do not show a direct impact of these adaptations for migraine patients: the percentage of attacks without a trigger remained constant (45% in wave 1 vs. 43.59% in wave 2), while the percentage of attacks without a symptom increased with almost 20% (0% in wave 1 vs. 19.23% in wave 2). On the contrary, in patients with CH, the adaptations of mBrain v2 resulted in no attacks without symptoms versus 45% of attacks in wave 1 patients (mBrain v1). It is important to readdress here that the lack of symptoms or triggers in wave 2 is explicit: for this the participants had to explicitly select the option “none of those” at the bottom of the list with available options. Moreover, while the average number of triggers per attack slightly increased from 0.75 to 0.83 for migraine patients, the average number of symptoms per attack even decreased from 4.40 to 2.29. This does not infer any concrete conclusion, since the selection of symptoms is still not explicit on an individual per-symptom basis. What can be learned from our experience is that explicit information about attack symptomatology or triggers is necessary in further app development.

The general statistics on headache attack registrations by migraine and CH patients seem to confirm existing knowledge about both disorder types. First, the number of attacks during a trial period of comparable length is higher for CH. Second, the attacks of CH patients are shorter. Third, the attack semeiology for both disorders is in concordance with medical literature (e.g., more restlessness and cranial autonomic symptoms (CAS) in patients with CH versus more hypersensitivity symptoms and nausea in patients with migraine). Fourth, if we calculate the total percentage of unilateral attacks, the attacks of CH patients are always unilateral, compared to only approximately 61% of the attacks of migraine patients.

An interesting observation, which does not correlate with the ICHD-3 criteria, is that the average intensity of attacks is not higher for CH patients compared to migraine patients, despite the fact that CH attacks are considered to be one of the most severe experiences of pain humans may have. A possible explanation was provided by a CH participant who stated that his or her assessment of the severity of attacks is subjective: due to desensitization, the patient assesses the severity lower compared to the period recently after the attacks started and the diagnosis was made. The observation that some CH patients label the severity of certain attacks as mild or moderate has already been documented by other authors [64, 65]. In fact, some migraine patients with attacks of mild pain intensity also mentioned this subjective assessment during the outtake visit, although attacks in migraine patients may also represent close to the phenotypical form of TTH and the final moments of a migraine attack may resemble more characteristics fitting the TTH criteria [66]. Lastly, it can be observed that the number of registered symptoms is lower for CH patients compared to migraine patients. However, both CH patients in wave 1 confirmed they did not (always) check all symptoms in the list when registering a headache.

The inquiry of headache symptoms and triggers in a smartphone application system is an example of finding the balance between not having too big of an impact on the participant’s daily life and routines, and making sure that the received information is as explicit as possible. The way that the information was requested in mBrain v1 was too focused on low intrusion, causing low information explicitness. Ideally, for every relevant symptom, the participant should indicate whether it is applicable or not with an explicit yes or no question. However, this would lean too much to the other side of the balance, potentially causing patients to not register any headache attacks as it becomes too time-consuming. Therefore, the changes in mBrain v2 try to find the right balance somewhere in the middle.

#### Classifications

Closely analyzing the classifications of the headache attacks registered by the participants of both data collection waves, some general observations can be made, as well as noteworthy findings about the specific disorders.

A first and important observation in all versions of the classification criteria, is the high number of headache attacks that are classified as *episodic TTH*. From the results in Table 12 we can calculate that with version 3 of the criteria, 89 out of 133 attacks (66.92%) receive this classification. 69 of those attacks (77.53%) also fulfill the required duration of 30 min to 7 days, while the other 20 attacks (22.47%) are all shorter than 30 min. However, a disclaimer should be made here. In version 3 of the classification criteria for *episodic TTH* in Table 3 (set (b)), many criteria require the *absence* of a certain symptom: *no* pulsating pain, *not* aggravated by routine physical activity, *no* nausea, *no* vomiting, *not* both photophobia and phonophobia. These symptoms are all present in the list of selectable symptoms upon the registration of a headache attack, as indicated in Table 2. As explained before, all unselected symptoms are implicitly considered to be non-applicable. Hence, a headache attack without any selected symptom will automatically fulfill the required classification criteria of *episodic TTH*. For version 1 and 2 of the criteria, this is true if the duration criterion is also fulfilled. In wave 1, selecting at least one symptom or selecting “none of those” was not yet required, and even in wave 2, no explicit “yes” or “no” answer is required for each individual symptom’s presence. Hence, incomplete registrations can lead to wrong classifications of *episodic TTH*. In contrast to *episodic TTH*, all symptom-related required classification criteria for *migraine without aura* and *CH* rely only on the *presence* of symptoms, which is always explicit. This is true for all versions of the classification criteria.

In general, observing the results of applying version 1 of the classification criteria to the registered headache attacks, the answer to the question whether the ICHD-3 diagnostic criteria can be strictly applied as classification criteria for individual attacks in a continuous follow-up setting, seems to be negative. In addition to the high number of attacks classified as *episodic TTH* episodes, the number of classifications as *migraine without aura* and *CH* is low: we can calculate from the results in Table 10 that out of all 98 headache attacks experienced by migraine patients, only 8 are classified as *migraine without aura*, and no attack as *CH*. For the 35 attacks experienced by CH patients, the results are even worse: no attack receives any of both classifications. This leads to the conclusion that this version of the algorithm is neither sensitive nor specific for the discriminatory task between migraine without aura and CH attacks on the one hand and TTH episodes on the other hand.

An important reason for this observation is the treatment criterion, which is often not fulfilled in patients who have acute headache treatment in place. Concretely, we can calculate from the statistics in Table 9 that approximately 66% of all attacks are treated and therefore could never be classified as *CH* if the treatment criterion would be taken into account. For *migraine without aura*, we can calculate that this number is 54%, since approximately 54% of all attacks are *successfully* treated. It is important to clarify that version 1 of the classification criteria was designed to strictly follow ICHD-3 definitions of headache attacks, even though ICHD-3 criteria for migraine and CH were designed to diagnose headache syndromes by analyzing multiple historic individual untreated attacks. There are currently no formal separate criteria in ICHD-3 for individual headache attacks only, in addition to the classification of disorders.

As such, the above discussed results from version 1 of the classification criteria confirm the rationale to test the exclusion of the treatment criterion in version 2 of the classification criteria. Observing the results of version 2 of the classification criteria, we can calculate that the number of *migraine without aura* classifications for attacks of migraine patients improve from 8 to 18. For CH patients, 6 out of the 35 attacks (17.14%) now receive the classification of *CH*. Because these numbers are still quite low, the criteria were further refined into version 3 of the criteria.

The main change to version 3 of the classification criteria was to also exclude the duration criterion, because of two reasons. First, ICHD-3 does not specify the required duration of a (successfully) treated attack. Second, the exact duration of an attack is often not *as* important as other location-related and symptom-related criteria. Observing the data, it is indeed true that there are attacks that fulfill those requirements, but not the duration requirement.

Taking a closer look at the classifications of the attacks of migraine patients with version 3 of the classification criteria, it can be calculated that only 28 of the 98 attacks (28.57%) are actually classified as *migraine without aura*. For this disorder, the required set of classification criteria in Table 3 consists of two distinct criteria, annotated with letter C and D as in ICHD-3. While 59 out of 98 attacks (60.20%) fulfill criterion C, only 34 of the 98 attacks (34.69%) fulfill criterion D. Hence, criterion D is the main limiting factor to not have the remaining attacks be classified as *migraine without aura*. This criterion D requires associated nausea, vomiting, or the combination of photophobia and phonophobia. As can be calculated from the results in Table 9, 38 out of 98 attacks (38.78%) have associated photophobia, 30 (30.61%) have phonophobia, 20 (20.41%) have nausea, and no attacks have associated vomiting. Hence, the low number of *migraine without aura* classifications is mainly caused by the lack of nausea or vomiting, and by the fact that often only one of photophobia or phonophobia occurs instead of both together. From the diagnostic criteria of ICHD-3, an attack fulfilling all but one criteria of *migraine without aura* only implies the diagnosis of *probable migraine without aura*, given no other ICHD-3 diagnosis is better accounted for. In general, this shows the difficulty that ICHD-3 has with capturing the intra-individual heterogeneity of migraine attacks into one set of criteria. This difficulty has an obvious impact on a system that assesses every attack individually, as compared to making a diagnosis based on a series of attacks. Therefore, further improving this classification system should consist of looking for techniques to incorporate these differences and trying to also integrate the *probable* disorder criteria in some way.

Interestingly, with version 3 of the classification criteria, we can calculate that 19 attacks of migraine patients are classified as *CH*. Observing the classification criteria of *CH*, two main components can be distinguished: (1) the pain should be unilateral around the orbit or temple, and (2) the pain should have at least one associated symptom out of a given set. Because of the high intra-individual heterogeneity of migraine attacks, the location criteria (1) are sometimes fulfilled: 66 out of 98 migraine attacks (67.35%) were unilateral, and 56 of them (84.85%) were in the orbital, supra-orbital or frontal head region as well. The set of symptoms (2) consists of restlessness/agitation, and the CAS. Previous research has shown that migraine and CH share common features in both the ICHD-3 criteria and semeiological descriptions [67–69], and that CAS regularly occur as symptoms of migraine attacks, even though they are not included in the ICHD-3 diagnostic criteria of migraine disorders [68]. Indeed, 34 of the 98 attacks experienced by the migraine participants had associated CAS. However, this previous research also shows that these CAS associated to migraine attacks are often bilateral, less severe, unrelated to the headache side, and less consistent with the headache attacks. Currently, the mBrain app allows no specification of such symptom characteristics. This would be especially relevant for the headache side, since the ICHD-3 criteria for *CH* require the CAS to be ipsilateral to the headache. Currently, it is implicitly considered that this is the case in the classification process. Future improvement of the headache registration process should therefore include the explicit request of this information. This will be especially useful for migraine patients who often experience unilateral headache attacks.

Moving over to the headache attacks experienced by CH patients, it is remarkable that 3 out of 20 attacks (15%) are classified as *CH* with version 3 of the classification criteria in wave 1, and 14 out of 15 attacks (93.33%) in wave 2. This is a dramatic improvement in classification accuracy. Part of this can be explained by the fact that the 2 CH patients in wave 1 did not register many symptoms: 9 of the 20 attacks (45%) do not have any associated symptom, and the other 11 attacks together have only 13 associated symptoms. Both patients confirmed that they did not explicitly check all symptoms upon the registration of a headache. Analysis indeed shows that it is mostly due to the symptoms that many attacks were not classified as *CH*: 17 of the 20 attacks (85%) were unilateral around the orbit or temple, fulfilling the non-symptom-related criteria. For the second wave, the results are much better, since 14 out of the 15 attacks experienced by the 2 CH patients are classified as *CH*. Moreover, the results suggest that not taking the registered severity as a requirement for the classification is a reasonable decision: out of the 35 attacks, only 7 (20%) have a severity of “severe”, and all other 28 attacks (80%) have a lower severity. As described earlier, this might be caused by a subjective lower assessment of the pain due to desensitization, as confirmed by one of the CH participants.

Finally, to come back to the *episodic TTH* classifications, this high number in CH patients (32/35, 91.43%) can also largely be explained by the lack of registered symptoms, as explained before. Only 14 of these attacks (43.75%) actually fulfill the duration criterion for *episodic TTH* (longer than 30 min), meaning the other 18 attacks (56.25%) are shorter than 30 min. From a biological viewpoint, this confirms that considering the duration for classification helps to distinguish between both, also because rapid treatment of CH attacks with oxygen or sumatriptan can result in attack abortion within minutes. Comparing this to *episodic TTH* classifications in migraine patients, the relatively high number there (57/98, 58.16%) cannot fully be explained by the lack of symptoms, since there are almost 3 symptoms on average associated to each attack. However, a possible explanation is the often-unfulfilled criterion D of the *migraine without aura* version 3 classification criteria in Table 3, since this criterion is the logical complement of criterion D in the required set of classification criteria for *episodic TTH*.

Overall, not considering the *episodic TTH* classifications and ignoring the participant with no registered headache attacks, 12 of the 17 remaining patients (70.59%) have mostly diagnosis classifications, i.e., the disorder type for which there are the most classifications out of the patient’s attacks, corresponds to the patient’s diagnosis. This number is already quite good, but the absolute number of *migraine* and *CH* classifications is still rather low. Further improvement of the presented preliminary classification system is therefore needed, by discussing and further shaping the classification criteria and the collected data that they are applied to.

A few limitations to this part of the study need to be addressed. First, we were only able to analyze a small number of participants in each group due to technical limitations of the study, i.e., limited available Empatica devices, spreading data load on server environment over time, and Empatica battery life issues. Second, also for technical reasons, the duration of the trial was only 21 days. The dynamic and cyclical nature of headache disorders often spans over multiple weeks, months or years. A 3-week period therefore does not seem enough to investigate a sufficient number of attacks to investigate their complexity and to develop personalized models for individual patients. It is the authors’ belief that a follow-up study should look into a minimum of 3 months of trial duration. Third, the participants were not asked to classify their attacks as either migraine without aura, CH or TTH. This was mainly because the objective of the set-up is to reflect the clinical reality as close as possible, by collecting clearly defined headache features by the subjects only and not self-diagnosing. We analyzed that subjects should not be qualified at this moment to provide a “ground truth” diagnosis of their individual attacks, because participants were not medically trained people and were not trained on the ICHD-3 criteria.

Moving forward in the development of an autonomous classification system for individual headache attacks, a few suggestions for improved systems can be derived from these results. First, more important than duration or treatment status to classify attacks are the symptoms of the attack which are a direct consequence of the underlying biological processes of each disorder. Second, because headache attacks within a headache syndrome can be heterogeneous intra-individually, the inclusion of new categories *probable migraine without aura* and *probable cluster headache* based on ICHD-3 guidance would be helpful to provide the clinician and patient a more nuanced and detailed overview of the different attacks. Third, because of the evolution of digital tools in medicine, it is our belief that there is a need for expert consensus within the international headache criteria to define specific criteria for different phenotypical types of headache attacks in concordance with the underlying biology of the disorders, to support longitudinal, momentary assessments in headache medicine for both clinicians and researchers. Such criteria should also consider having separate criteria based on the treatment criterion which currently limits the number of attacks for classification as shown in our results.

An important path to follow in the future improvement of the classification criteria, is the investigation of the possible inclusion of the contextual data that is collected during the mBrain study. Up to now, the classification is purely based on the static information that is entered by the patient when registering a headache attack in the mBrain app. However, much more data is available to use for classification. A potentially important source are the activity, stress and sleep events that are continuously generated by the data-driven ML algorithms, based on the physiological data collected with the Empatica E4 wearable. From this and other data, some symptoms or triggers could be measured and validated. One example may be activity or movement measurements derived from wearable data, since CH attacks are mostly characterized by restlessness and agitation while migraine patients tend to withdraw from activities before and during attacks [6, 7]. Hence, it will be important to research, in close collaboration with headache experts, whether such contextual data can improve the classification results.

### Data collection and interaction with automatically added events

Given the objectives of the mBrain study, the collected data should be as complete and as accurate as possible. Therefore, different parameters are important: the amount of data that is being collected, the interaction rate of patients with the applications determining the amount of feedback, and the accuracy of the data-driven ML algorithms. These aspects are analyzed and discussed below based on the general statistics of the data collection presented in Table 8. An important aspect of this analysis is the impact of the changes implemented into the second wave as explained in the “mBrain v2’’ section.

#### Amount of data collected

The amount of time that the participants collected data with their Empatica device was smaller than expected during the first wave, with a little over 9 h on average, even though participants are requested to wear the Empatica during both day and night. There are large differences between the participants, from more than 15  h to only 5 h of data on average per day. Different reasons were given by the participants: some found it difficult to integrate the procedure into their daily routine, while others struggled to keep their smartphone closeby (i.e., to their Empatica) all the time, causing the device to frequently disconnect. A third reason was difficulty dealing with the—sometimes shorter than expected—battery lifetime, requiring the patient to charge the battery multiple times per day. Unfortunately, these reasons did not allow for straightforward adaptations to the data collection process. Ideally, an automatic reconnection mechanism would be available that eliminates the need to manually reconnect the Empatica every time the connection is interrupted. This is something that will be available in the next Empatica SDK, which is not scheduled for release yet by Empatica. The successor of the Empatica E4 will also allow for on-device charging without interrupting the Bluetooth connection, which would also decrease the impact of battery issues. For now, it can be observed from the results of the second wave that the average connected time did increase with over 3.5 h only thanks to stressing the importance of collecting more data during the intake visit. This is already better, but still leaves room for improvement.

Moreover, the impact of adapting the configuration of the OwnTracks app as described in the OwnTracks section, is also visible in the results: there is an increase of more than 18 location points on average per patient per trial day, indicating that the participants’ location was followed up more closely during the second wave.

Finally, one patient did not register any headache attacks throughout the trial period. This highlights the fact that recruiting patients in their active headache period is crucial. Having no headache data about a patient means that it is not possible to find any relations, making the patient’s participation less useful.

#### Level of interaction with automatically added events

The interaction rate with the automatically added events in the timeline was quite low during the first data collection wave: on average 71% of the activity events, 88% of the sleep events and 73% of the stress periods were never interacted with. However, it is important to have explicit feedback about this contextual information. To this end, as many events as possible should either be confirmed, corrected or removed.

Two main reasons for this were identified during the outtake visits: recall bias, and the large number of events per day in combination with a lack of time to process them all. Therefore, the decision was made to implement changes to the timeline in mBrain v2: a default normal timeline view with a significant reduction in number of activity events by merging sedentary activities, a restriction on the number of stress events per hour and day, and a notification about each stress event to make the patient interact with it as soon as possible and reduce possible recall bias. Moreover, during the second data collection wave, participants were stressed even more to interact and provide explicit feedback as often as possible.

The impact of the adaptations to the timeline of mBrain v2 is clearly visible in the results. The absolute average percentage of automatically added activities that were ignored decreased with more than 45%, while for stress events this decrease was more than 49%. Also for sleep events, there was an absolute decrease of more than 28%. These results demonstrate the relevance and importance of further improving these and other features that might influence the amount and accuracy of the feedback received from the participants.

Moreover, the confirmation and removal of stress events is another example of where the balance between daily life intrusion and information explicitness needs to be found. If an automatically added stress event was removed during the first wave, it was unclear why. This is in contrast to activities or sleep, where the type of activity can always be corrected, allowing for more explicit feedback. Similarly, for confirmed stress events, it was never known what the stress intensity was, since participants were not forced to edit the event and enter this intensity. Because this information is important for the further improvement of the data-driven stress detection algorithm, small additional questions were asked in mBrain v2 upon confirming or removing a stress event. In other words, the requested effort of the patient was slightly increased, in return for some more explicit feedback. Looking at the results of the second wave, the number of confirmations did not drop because of the additionally requested input, but instead largely increased from less than to 2% to almost 45%. The number of deletions of stress events did decrease with more than 2% on average in wave 2 compared to wave 1, but the number of corrections to stress events with level 0 increased with on average more than 11%. As such, it is difficult to assess the correlation between the newly requested input and these changes in the numbers.

#### Outcome of interactions with automatically added events

To assess how well the data-driven ML algorithms can map the patient’s activities, stress and sleeping behavior, the automatically added timeline events that the patients *have* interacted with are analyzed, especially for the results of the second data collection wave. In this wave, many improvements were introduced, which positively influenced the amount of feedback received.

For the activities that were interacted with during the second data collection wave (on average 74%), on average almost 46% of the predicted activities were fully confirmed, i.e., with an explicitly confirmed type. In addition, 12% on average were confirmed as sedentary in the new normal timeline view. Less than 6% on average were corrected, and the remaining 10% were removed. First, this shows that the predictions of the activity recognition algorithms are correct in most times. Second, these results show the benefit of splitting up the timeline in two views, allowing for fine-grained or coarse-grained feedback depending on the available time of the participant. Especially since on average 88% of activities were of a sedentary type (in terms of number of events, not considering duration), the number of events in the normal timeline view was significantly reduced by merging them where possible.

For the stress events that were interacted with during the second data collection wave (on average 76%), approximately 59% of them (45% of all stress events) were confirmed with a moderate or high stress intensity, while the others were either removed or corrected to an event with stress intensity 0, which are semantically equivalent. These results are already way better than during the first wave, where only 4% of the stress events were confirmed, but there is still room for improvement.

Finally, for sleep events, approximately one third of the interactions on average was a confirmation. However, patients seemed to register their sleep periods more manually compared to activity or stress events. This could possibly be explained by two reasons. First, the sleep algorithm runs only once every 24 h, causing these events to not be present yet in the timeline in the mornings. Second, the sleep algorithm requires physiological data from the Empatica throughout the full sleeping period to detect it. Given the average amount of Empatica data per day, some sleeping periods might therefore have not been detected.

In conclusion, it is clear that the algorithms are already able to map the patient’s activities, sleep and stress reasonably well, but that further improvement of them will remain crucial. In a next phase, an interesting path to investigate is the personalization of the individual predictive models, per patient.

Translating the mBrain set-up into the real world would decrease the expected user burden because of several reasons. First, interacting with the events predicted by the ML models would no longer be required as they should be accurate enough. An easy headache attack registration process, e.g., by hitting the event button of the Empatica, would lead to a decreased registration burden. Moreover, on-device charging and automatic BLE reconnection with temporary buffering are expected future features of new Empatica devices and their SDKs, which will lead to a higher amount of collected wearable data. Finally, it should be noted that the machine-learning algorithms are generic and device-independent, meaning the Empatica could easily be replaced by another wearable that measures the same physiological data.

### Knowledge-based detection of headache triggers

The evaluation with the example cases, demonstrated in the “Knowledge-based trigger detection results” section, shows that at least for physical exercise and stress, the indication by a patient of specific triggers for an attack, can be observed from these contextual events in some cases. In this evaluation, the system uses the triggers indicated by a patient to retrospectively check the data collected in the period before that headache. However, for a trigger detection system to work, triggers need to be known upfront. This is not unrealistic. If a certain event is a trigger for a headache attack, it is not unlikely that it will be a trigger for future attacks as well. During the intake visit of patients, the physician-researcher could therefore integrate questions specifically targeted at querying frequently occurring triggers. Moreover, by investing in the data-driven learning of triggers for patients based on the collected data, new triggers could be discovered, potentially including triggers that the patient is not (yet) aware of himself. In the latter case, sending a trigger alarm could be especially relevant.

For the concrete design of the individual trigger detection queries in such a system, more research is needed concerning how to define triggers, how to detect each trigger based on the available data, the optimal personalized time window, among other things. Also, collecting other contextual data could enlarge the set of detectable headache triggers. An example could be the detection of flickering light or loud noise through the collection of light intensity and noise data.

While researching those new systems, it should not be forgotten, however, that these “triggers” may also be a misconception of the presence of premonitory symptoms already happening before the trigger and headache attack occur [61]. For example, chocolate may not be a trigger for migraine but rather the craving towards sweets may be a premonitory symptom already present before the patient eats chocolate [70].

In summary, the fact that currently indicated triggers can often be backed up with the collected data, proves the potential usefulness of a trigger detection system. In addition, it is another example of why it is important and useful to invest time and resources into the collection of a wide range of physiological and contextual data through the Empatica E4 wearable and the various applications, and the design of data-driven algorithms that analyze this data to detect certain events, in order to improve the continuous follow-up of headache patients.

## Conclusions

In this paper, the set-up and first results of the mBrain study are discussed. mBrain is an exploratory, observational research study that investigates how to move from the intermittent, subjective follow-up and classification of headaches based on self-reported data, towards a more continuous, semi-autonomous, objective follow-up and classification that is based on a combination of self-reported data, and objective physiological and contextual data. Therefore, physiological data is automatically collected with the Empatica E4 wearable. Data-driven ML algorithms use this data to detect the activities, stress events and sleeping behavior of the patients. Using a mobile application, patients can interact with these events, and keep a diary of other contextual and headache-specific data.

As a first subquestion, the study has investigated how to collect as much objective and explicit data as possible about a patient’s headache attacks and relevant context. After a first data collection wave, several changes implemented into the set-up have successfully improved the level and accuracy of the received feedback on predictions of the ML algorithms during a second data collection wave. This shows that it is relevant to keep further improving and fine-tuning this set-up, while balancing between daily life intrusion and information explicitness, to obtain a complete and correct view on the patient’s context and lifestyle.

Second, the paper has researched how to design an autonomous classification system for individual headache attacks. Therefore, a knowledge-based system was designed to classify registered attacks as either migraine without aura, CH, or episodic TTH. Different versions of classification criteria were designed, starting from the ICHD-3 diagnostic criteria. The results show that strictly applying the ICHD-3 criteria on individual attacks does not yield good classification results. Adapted versions yield better results, leading to mostly diagnosis classifications for 12 of the 18 patients if episodic TTH classifications are ignored. However, the absolute number of migraine without aura (28/98) and CH classifications (17/35) is still rather low. Therefore, further shaping the classification criteria and data they are applied to is required. An interesting path to investigate here is whether and how the events detected by the ML algorithms can be integrated into the classification process. Moreover, specifically for migraine patients, it should be further researched how to deal with the intra-individual heterogeneity of migraine attacks.

Third, to integrate the output of the data-driven ML algorithms for the continuous follow-up and classification of headache attacks, it should present an accurate view on the patients’ context. The results of the second data collection wave show that this is largely true for activity events, and that serious improvements have been made for stress and sleep events. Therefore, further refinement of the different algorithms will remain important. It should be investigated whether the personalization of the individual predictive models can increase the overall accuracy.

Fourth and final, the paper has taken the first steps to investigate how the physiological, contextual and headache-related data of patients can be linked to be valuable for the continuous follow-up of headaches. To this end, two example cases have demonstrated the potential of using the outputs of the data-driven ML algorithms for the knowledge-based detection of known headache triggers. In addition, it will be useful to research how headache triggers for specific patients can be discovered by data-driven learning techniques. In summary, this highlights the potential of focusing on hybrid AI for the future improvement of continuous headache follow-up, classification and trigger detection.

## Supplementary Information

**Additional file 1.** General-data-collection-statistics. General statistics of the first and second data collection wave of the mBrain study. These statistics are complementary to Table 8 of the main manuscript: the same statistics are shown, complemented by spread measures where appropriate, in two separate tables (one per wave). The statistics include data collection statistics, timeline activity & interaction statistics, and daily record statistics.

## Data Availability

The datasets generated during and/or analyzed during the current study are not publicly available, as imposed by the Ethics Committee of the Ghent University Hospital due to the protection of privacy of the participants. The DAHCC ontology is publicly available at https://github.com/predict-idlab/DAHCC-Ontology.

## Abbreviations

API: Application programming interface
BLE: Bluetooth Low Energy
BVP: Blood volume pulse
CAS: Cranial autonomic symptoms
CH: Cluster headache
DAHCC: Data Analytics for Health and Connected Care
EDA: Electrodermal activity
GBD: Global Burden of Disease
GSR: Galvanic skin response
HR: Heart rate
IBI: Inter beat interval
ICHD-3: The International Classification of Headache Disorders, Third Edition
IHS: International Headache Society
IQR: Interquartile range
MIDAS: Migraine Disability Assessment Test
ML: Machine learning
MSQ: Migraine-Specific Quality-of-Life Questionnaire
PPG: Photoplethysmogram
RDF: Resource Description Format
SD: Standard deviation
SF-20: MOS Short-Form General Health Survey
SPARQL: SPARQL Protocol and RDF Query Language
TTH: Tension-type headache
WHO: World Health Organization
