# Supplementary material for: mBrain: towards the continuous follow-up and headache classification of primary headache disorder patients
Source: BMC Med Inform Decis Mak. 2022 Mar 31;22:87. doi: 10.1186/s12911-022-01813-w (PMC8969243; doi:10.1186/s12911-022-01813-w)
Supplement: Supplementary file 1 — Additional file 1. General-data-collection-statistics. General statistics of the first and second data collection wave of the mBrain study. These statistics are complementary to Table 8 of the main manuscript: the same statistics are shown, complemented by spread measures where appropriate, in two separate tables (one per wave). The statistics include data collection statistics, timeline activity & interaction statistics, and daily record statistics. [file 12911_2022_1813_MOESM1_ESM.pdf]

Supplementary table 1 General statistics of data collection during the first mBrain data collection wave. Statistics mentioning "(pp)" are "per patient" statistics: they are first calculated per patient, and then aggregated over all patients. Other abbreviations used in this table: aut. is automatically added, man. is manually added, pct. is percentage, SD is standard deviation, pds. is periods, w/ is with, w/o is without, '#' represents 'number of'.

	Wave 1
# patients	7
Duration of trial (days) (pp), mean (SD)	22.57 (1.62)
Data collection statistics	
Connected Empatica time per trial day (HH:mm) (pp), mean (SD)	09:06 (03:54)
# location points per trial day (pp), mean (SD)	91.49 (151.62)
# tags per trial day (pp), mean (SD)	0.41 (0.56)
Timeline activity & interaction statistics	
Headache attacks	
# headache attacks (pp), mean (SD)	5.71 (4.68)
Pct. of patients w/o any headache attack	14.29%
Medicine intakes	
# medicine intakes (pp), mean (SD)	3.86 (5.67)
Pct. of patients w/o any medicine intake	57.14%
Activities	
# man. activities (pp), mean (SD)	1.07 (0.46)
# aut. activities per trial day (pp), mean (SD)	35.81 (12.71)
Pct. of aut. activities of sedentary type (pp), mean (SD)	90.42% (3.47%)
Pct. of aut. activities *fully* confirmed (pp), mean (SD)	19.19% (26.72%)
Pct. of aut. activities *only* confirmed as sedentary, but w/o explicit confirmation of predicted type (pp), mean (SD)	0.00% (0.00%)
Pct. of aut. activities w/ *only* corrected time (pp), mean (SD)	0.04% (0.08%)
Pct. of aut. activities w/ corrected type (pp), mean (SD)	6.34% (6.83%)
Pct. of aut. activities removed (pp), mean (SD)	3.22% (7.42%)
Pct. of aut. activities ignored (pp), mean (SD)	71.20% (37.99%)
Sleep periods	
# man. sleep pds. (pp), mean (SD)	13.86 (5.70)
# aut. sleep pds. (pp), mean (SD)	31.57 (9.91)
Pct. of aut. sleep pds. confirmed (pp), mean (SD)	6.11% (9.98%)
Pct. of aut. sleep pds. w/ corrected time (pp), mean (SD)	0.48% (1.26%)
Pct. of aut. sleep pds. corrected to activity (pp), mean (SD)	0.00% (0.00%)
Pct. of aut. sleep pds. removed (pp), mean (SD)	5.46% (8.33%)
Pct. of aut. sleep pds. ignored (pp), mean (SD)	87.96% (16.08%)
Stress periods	
# man. stress pds. per trial day (pp), mean (SD)	0.04 (0.08)
# aut. stress pds. per trial day (pp), mean (SD)	15.86 (11.30)
Pct. of aut. stress pds. confirmed w/o level (pp), mean (SD)	2.63% (5.92%)
Pct. of aut. stress pds. confirmed w/ level 1/2 (pp), mean (SD)	1.48% (3.70%)
Pct. of aut. stress pds. corrected w/ level 0 (pp), mean (SD)	17.49% (29.45%)
Pct. of aut. stress pds. removed (pp), mean (SD)	5.00% (6.17%)
Pct. of aut. stress pds. w/ corrected time (pp), mean (SD)	0.03% (0.07%)
Pct. of aut. stress pds. ignored (pp), mean (SD)	73.37% (36.33%)
Daily record (DR) statistics	
Pct. of trial days w/ part of DR provided (pp), mean (SD)	78.89% (19.00%)
Pct. of trial days w/ daily stress level provided (pp), mean (SD)	73.07% (25.05%)
Pct. of trial days w/ daily mood provided (pp), mean (SD)	73.66% (23.75%)
Pct. of trial days w/ daily food intake provided (pp), mean (SD)	60.17% (28.33%)
Pct. of trial days w/ DR fully completed (pp), mean (SD)	58.87% (30.68%)

Supplementary table 2 General statistics of data collection during the second mBrain data collection wave. Statistics mentioning "(pp)" are "per patient" statistics: they are first calculated per patient, and then aggregated over all patients. Other abbreviations used in this table: aut. is automatically added, man. is manually added, pct. is percentage, SD is standard deviation, pds. is periods, w/ is with, w/o is without, '#' represents 'number of'.

	Wave 2
# patients	11
Duration of trial (days) (pp), mean (SD)	22.09 (0.30)
Data collection statistics	
Connected Empatica time per trial day (HH:mm) (pp), mean (SD)	12:49 (04:49)
# location points per trial day (pp), mean (SD)	109.78 (83.84)
# tags per trial day (pp), mean (SD)	0.89 (1.07)
Timeline activity & interaction statistics	
Headache attacks	
# headache attacks (pp), mean (SD)	8.45 (3.14)
Pct. of patients w/o any headache attack	0.00%
Medicine intakes	
# medicine intakes (pp), mean (SD)	5.64 (3.38)
Pct. of patients w/o any medicine intake	9.09%
Activities	
# man. activities (pp), mean (SD)	2.60 (1.77)
# aut. activities per trial day (pp), mean (SD)	46.24 (16.16)
Pct. of aut. activities of sedentary type (pp), mean (SD)	88.29% (6.22%)
Pct. of aut. activities *fully* confirmed (pp), mean (SD)	45.90% (30.46%)
Pct. of aut. activities *only* confirmed as sedentary, but w/o explicit confirmation of predicted type (pp), mean (SD)	12.01% (14.83%)
Pct. of aut. activities w/ *only* corrected time (pp), mean (SD)	0.56% (0.64%)
Pct. of aut. activities w/ corrected type (pp), mean (SD)	5.31% (6.03%)
Pct. of aut. activities removed (pp), mean (SD)	10.27% (8.31%)
Pct. of aut. activities ignored (pp), mean (SD)	25.94% (33.73%)
Sleep periods	
# man. sleep pds. (pp), mean (SD)	19.45 (6.74)
# aut. sleep pds. (pp), mean (SD)	37.36 (13.17)
Pct. of aut. sleep pds. confirmed (pp), mean (SD)	13.46% (13.16%)
Pct. of aut. sleep pds. w/ corrected time (pp), mean (SD)	2.62% (3.76%)
Pct. of aut. sleep pds. corrected to activity (pp), mean (SD)	0.70% (2.32%)
Pct. of aut. sleep pds. removed (pp), mean (SD)	23.43% (28.16%)
Pct. of aut. sleep pds. ignored (pp), mean (SD)	59.79% (30.52%)
Stress periods	
# man. stress pds. per trial day (pp), mean (SD)	0.05 (0.11)
# aut. stress pds. per trial day (pp), mean (SD)	6.66 (2.84)
Pct. of aut. stress pds. confirmed w/o level (pp), mean (SD)	0.00% (0.00%)
Pct. of aut. stress pds. confirmed w/ level 1/2 (pp), mean (SD)	44.89% (32.41%)
Pct. of aut. stress pds. corrected w/ level 0 (pp), mean (SD)	28.62% (28.03%)
Pct. of aut. stress pds. removed (pp), mean (SD)	2.66% (2.58%)
Pct. of aut. stress pds. w/ corrected time (pp), mean (SD)	0.04% (0.14%)
Pct. of aut. stress pds. ignored (pp), mean (SD)	23.79% (34.77%)
Daily record (DR) statistics	
Pct. of trial days w/ part of DR provided (pp), mean (SD)	92.99% (6.55%)
Pct. of trial days w/ daily stress level provided (pp), mean (SD)	91.34% (6.59%)
Pct. of trial days w/ daily mood provided (pp), mean (SD)	91.34% (6.59%)
Pct. of trial days w/ daily food intake provided (pp), mean (SD)	87.62% (6.15%)
Pct. of trial days w/ DR fully completed (pp), mean (SD)	87.62% (6.15%)
